# Roadmap of Terahertz Imaging 2021

**DOI:** 10.3390/s21124092

**Published:** 2021-06-14

**Authors:** Gintaras Valušis, Alvydas Lisauskas, Hui Yuan, Wojciech Knap, Hartmut G. Roskos

**Affiliations:** 1 Center for Physical Sciences and Technology (FTMC), Department of Optoelectronics, Saulėtekio Ave. 3, LT-10257 Vilnius, Lithuania; 2 Institute of Photonics and Nanotechnology, Department of Physics, Vilnius University, Saulėtekio Ave. 3, LT-10257 Vilnius, Lithuania; 3 Institute of Applied Electrodynamics and Telecommunications, Vilnius University, Saulėtekio Ave. 3, LT-10257 Vilnius, Lithuania; alvydas.lisauskas@ff.vu.lt; 4 CENTERA Laboratories, Institute of High Pressure Physics PAS, Sokolowska 29/37, 01-142 Warsaw, Poland; knap.wojciech@gmail.com; 5 Physikalisches Institut, Goethe-Universität, Max-von-Laue Straße 1, D-60438 Frankfurt am Main, Germany; yuan@physik.uni-frankfurt.de (H.Y.); roskos@physik.uni-frankfurt.de (H.G.R.)

**Keywords:** teraherz imaging systems, terahertz emission, terahertz sensing, diffractive optics, teraherz nano-imaging and nanoscopy, computational imaging, on-chip solutions, artificial intelligence, applications of teraherz imaging

## Abstract

In this roadmap article, we have focused on the most recent advances in terahertz (THz) imaging with particular attention paid to the optimization and miniaturization of the THz imaging systems. Such systems entail enhanced functionality, reduced power consumption, and increased convenience, thus being geared toward the implementation of THz imaging systems in real operational conditions. The article will touch upon the advanced solid-state-based THz imaging systems, including room temperature THz sensors and arrays, as well as their on-chip integration with diffractive THz optical components. We will cover the current-state of compact room temperature THz emission sources, both optolectronic and electrically driven; particular emphasis is attributed to the beam-forming role in THz imaging, THz holography and spatial filtering, THz nano-imaging, and computational imaging. A number of advanced THz techniques, such as light-field THz imaging, homodyne spectroscopy, and phase sensitive spectrometry, THz modulated continuous wave imaging, room temperature THz frequency combs, and passive THz imaging, as well as the use of artificial intelligence in THz data processing and optics development, will be reviewed. This roadmap presents a structured snapshot of current advances in THz imaging as of 2021 and provides an opinion on contemporary scientific and technological challenges in this field, as well as extrapolations of possible further evolution in THz imaging.

## 1. Introduction

The first terahertz (THz, 1 THz is 10${}^{12}$ Hz) images recorded by T. S. Hartwick and co-workers in 1976 [1] using optically pumped molecular THz laser, later followed a seminal work by B. Hu and M. Nuss, demonstrating principles of optoelectronic THz imaging using femtosecond lasers-based sources in 1995 [2] have ever since induced an exciting burst of related activities. THz imaging science and technology experienced tremendous progress in the last two decades, both in regard to fundamental research and versatile applications [3,4,5]. THz imaging remains a fruitful field for the birth of new ideas while also bridging distinct developed technologies and transforming them into novel applications. This stems from the specific occupancy of THz frequencies in the electromagnetic radiation spectrum; they extend from 10${}^{11}$ Hz to 10${}^{13}$ Hz frequencies (corresponding energies are 0.4–40 meV), thus falling between the millimeter waves and the infrared range. In regard to device physics, it is the energy region between a classical carrier transport with the cut-off frequency defined mainly by carrier transit time or parasitic RC time constants towards the red end of the spectrum, and by quantum mechanics rules governed photonic devices towards the blue side. Since the THz energy window is very narrow, realization of population inversion conditions at room temperature is hard to achieve. In regard to applications, THz imaging experiences a growing interest due to unique ability of THz radiation to penetrate through many non-conducting materials, such as clothing and common packaging substances, thus offering a non-destructive way for imaging and spectroscopy [6,7]. Since many chemical and biological agents display characteristic spectral signatures within this range, THz devices open a new possibilities for inspection and quantification of these agents [8,9,10], also new materials [11,12] and even cultural heritage objects [13,14]. In addition, 1 THz corresponds merely to a quantum of 4.14 meV; thus, THz radiation is non-ionizing and non-invasive in contrast to X-rays used commonly for medical aims [15,16,17].

In this roadmap article, we are focusing on several recent advances in THz imaging. Particular attention is paid to optimization and miniaturization of the THz imaging systems; these solutions are of prime interest for direct technology implementation providing a sought-after convenience in real operational conditions. The most important achievements and contemporary scientific and technological challenges will be decomposed in this article with respect to the main system component—solid-state-based THz emitters, compact flat optics, THz room temperature detectors and their arrays, and miniaturized THz imaging systems with reduced power consumption. Special attention will be dedicated to cover achievements in room temperature frequency difference operational scheme-based quantum cascade lasers, fiber femtosecond lasers pumped THz imaging systems and evolution of compact electronic sources. Another focus area is dedicated to advanced room temperature solid-state THz sensors and their arrays, as well as their possible on-chip integration with diffractive THz optical components. A significant emphasis is attributed to beamforming role in THz imaging, THz holography and spatial filtering, THz nano-imaging and nanoscopy, and computational imaging. Several advanced THz techniques will be described, including light-field THz imaging, homodyne spectroscopy and phase sensitive spectrometry, THz modulated continuous wave imaging, room temperature THz frequency combs, and passive THz imaging. The implementation of artificial intelligence in THz imaging will be also outlined; finally, systems integration and possible future directions in THz imaging field will be envisaged.

## 2. Compact Solutions in THz Emitters

Optoelectronic THz time-domain (TDS) systems [18] were the main driving force in development of both THz spectroscopic tools and imaging approaches. The technique is based on the sampling of THz field with a femtosecond laser pulse; hence, it measures the transient electric field rather than its intensity [18]. THz TDS advantages relying on fast, sensitive, and coherent detection ability, as well as high signal-to-noise ratio, SNR, (i.e., ratio of the level of a measured signal to the level of the background noise), together with a rapid evolution of femtosecond lasers have stimulated extensive investigation dedicated for search of suitable materials and novel generation/detection principles. It was revealed that different physical processes—photocurrent surge effect due to the strong surface electric field induced band bending, the so-called photo-Dember effect, because of the spatial separation of the electrons and the holes due to their different mobilities, resulting in appearance of the electric field, second-order or the third-order susceptibility-related optical rectification or electrical field-induced optical rectification, respectively—can be found as responsible THz emission mechanisms in various semiconductors, such as radiation-damaged silicon-on-sapphire and low-temperature-grown, or ion-implanted GaAs, InGaAs, InAs, or GaAsBi semiconducting compounds [19]. Regarding investigated materials, one can note that the studies were not limited to semiconductors only; a special attention gained optical rectification-based THz emission from lithium niobate [20,21] and second-order nonlinear effects for THz generation in different type of organic crystals, like DAST, DSTMS, OH1, HMQ-TMS, and BNA [22,23,24,25,26]. It is worth noting air-plasma-based THz photonics approach when a high intensity femtosecond laser can induce plasma oscillations in air [27,28], which can emit broadband THz radiation [29,30]. However, despite the fact that aforesaid systems contain beautiful physics and can serve as powerful instruments for THz spectroscopy, they are relatively bulky, and they require specific and complicated set-up arrangements. As our focus is compact imaging, therefore, we will not discuss these approaches in more detail but will further restrict ourselves mainly in optoelectronic solutions enabling development of convenient-in-use THz imaging systems.

### 2.1. Fiber Femtosecond Laser-Based THz Sources

**Figure 1:**
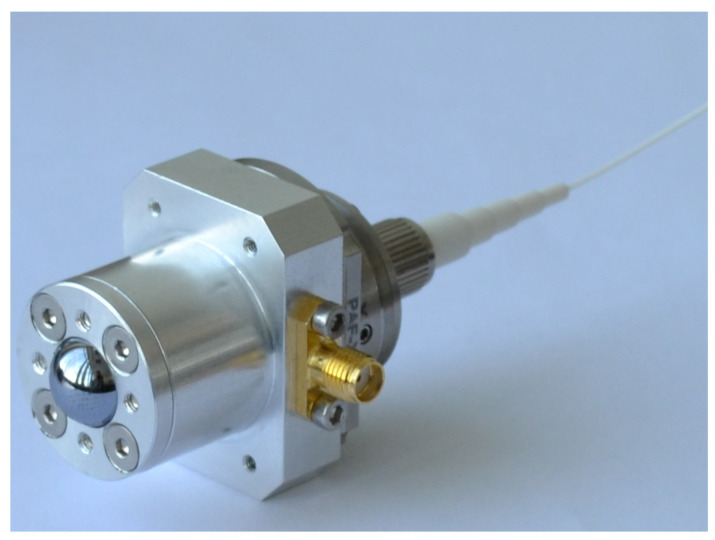
Photo of fiber-coupled optoelectronic GaAsBi-based THz emitter mounted with a silicon lens. Courtesy of Laboratory of Ultrafast Optoelectronics Laboratory at Optoelectronics Department at FTMC and Teravil Ltd., Vilnius, Lithuania.

*Recent advances*: Optoelectronic THz time-domain system successfully served in pioneering THz imaging experiment [2]. First, THz time-domain and imaging systems were bulky and mainly oriented to scientific laboratory uses; however, their recording principle, as mentioned above, relying on phase-locked detection enables very sensitive detection despite relatively low emission powers ranging below 1 µW. Conventional Ti:sapphire laser-based systems are also rather bulky, and they require a complex optical pumping scheme for their operation. The reduction of dimensions, the power consumption and user-friendly features can be achieved by replacing them, for instance, with ultrafast fiber laser-based systems. However, in this case, low-temperature grown GaAs become not suitable for THz generation/detection because of the change in the excitation wavelength, and it stimulates a search to design and fabricate suitable materials exhibiting short carrier lifetimes and large dark resistivities. One of the first solutions was to employ InGaAs/InAlAs photoconductive layers which allowed achievement of order-of-magnitude improvements versus planar antennas in terms of emission power, dark current and receiver sensitivity [31]. They were applied for continuous wave THz generation at 1.5 µm [32] optical pumping and in TDS using 1 µm wavelength excitation [33]. However, the structures were multi-layered, consisted of 100 periods InGaAs/InAlAs, and, thus, were technologically demanding and expensive for the task at hand. As a promising alternative was found to be based on ternary GaAsBi structures which were initially applied for the THz detection in TDS system using *p*-InAs as THz emitter [34]. Later, a TDS system was demonstrated based on Yb:femtosecond lasers and composed solely of GaAsBi materials for emission and detection [35]. Since GaAsBi grows intrinsically of *p*-type, it was not suited for 1.55 µm wavelength excitation. To overcome this, molecular beam epitaxy (MBE) synthesis of intrinsic of *n*-type InGaAs alloyed with Bi content was proposed and successfully demonstrated (Figure 1) in a TDS system using 1.55 µm pulses emitted by an Er-doped fiber laser [36]. The frequency limit of this system was found to be 4.5 THz, with 65 dB SNR ratio and the emission power reaching 5 µW. These studies also indicated sensitivity of the proper materials design for effective THz emission in various types of TDS systems.

Another recently study announced promising approach relies on MBE grown rhodium (Rh) doped InGaAs which exhibits a combination of ultrashort trapping time, high electron mobility, and high resistivity [37]. Because of these features, fiber coupled photoconductive THz emitters exhibit impressive characteristics: They delivered THz power amounts to 637 µW, the bandwidth extends up to 6.5 THz, and a record peak dynamic range reaches a value of 111 dB.

*Current challenges and possible future trends*: One can identify two key areas that need to be addressed for further optimization of fiber laser-based THz TDS systems.

The first one is related to materials engineering and the need to reach higher emission power levels, since the aforementioned values in the µW range cannot be found well-suitable for wide range of THz imaging implementations. Recent novel approaches employing additional mechanisms, such as nanoplasma, enabling switches to amplify optoelectronically generated THz emission [38], can gain a particular attention because of of their ability to reach impressive 600 mW power emission.

The second area concerns the need for effective antenna design aiming to extend the emission bandwidth of the THz TDS system for versatile spectroscopic measurements and its implementation in multispectral THz imaging systems.

Finally, deserving of mention is the optical light-to-THz radiation conversion efficiency aspect; longer wavelengths typically lead to more effective conversion. Development of Tm:YAG-based femtosecond lasers and engineering of THz source material, for instance, quaternary GaInAsBi alloy layers with with 6% Bi epitaxially strained to InP substrates, can be a route to a new optoelectronic THz systems using optical excitation pulses with wavelengths longer than 2 µm [39].

### 2.2. THz Quantum Cascade Lasers

*Recent advances*: Quantum cascade lasers (QCLs) are compact THz emission sources relying on optical transitions between electronic states created by engineering of electronic wavefunctions in MBE grown semiconductor nanostructures [40]. The QCLs experienced an intensive evolution over their more than 25 years of existence from an attractive laboratory proof-of-principle to a powerful technology in many versatile applications, such as trace gas analysis, optical communications, and real-time imaging [41,42].

Terahertz QCLs, invented in 2002 [40], have likewise undergone development with important milestones along the way. One of the most challenging issues in comparison to conventional infrared QCLs is reaching room temperature operation conditions and tunability of the devices for their usage in spectroscopy or multispectral THz imaging [43,44]. By varying the design of the constituent quantum structures, tunability within 1–5 THz can be achieved; concerning the emission power, it can exceed 1 W level in pulsed mode operation at 10 K and more than 100 mW in continuous wave regime at 3.4 THz frequency [45]. However, the highest operating temperature was determined to be of 123 K in these experiments. Even more encouraging numbers in power can be reached in THz quantum-cascade vertical-external-cavity surface-emitting laser by employing an amplifying metasurface designed for the increased power density: Metasurface composed of a sub-wavelength array of metal-metal waveguide antenna-coupled sub-cavities loaded with a THz quantum-cascade gain material allows for providing peak THz output powers up to 830 mW at 77 K and 1.35 W at 6 K in a pulsed mode operation, still maintaining a single-mode spectrum and a low divergence beam pattern [46]. The use of the phase-locking scheme of THz plasmonic QCLs enables to reach the measured peak output power in excess of 2 W for a single-mode 3.3 THz QCL radiating in a narrow single-lobed beam when operated at 58 K in a compact Stirling cooler [47].

Thermoelectric cooling is important in these applications to achieve a needed device working temperature. An important milestone in this direction is the demonstration of THz QCL emitting radiation around 3.9 THz at 210 K temperature with the use of a small footprint, 4-stage Peltier cooler [48]. It was the first operation of a THz QCL using thermoelectric cooling and the first operation of a THz QCL above 200 K, with a peak power as high as 1.2 mW at 206 K, which allowed detection in a cryogenic-free system. Very recently, another important milestone was demonstrated with THz QCL operating at 4 THz with a maximum operating temperature of 250 K [49]. The device is very compact—only the size of a rice grain—and, thus, opens a promising route to develop portable on-chip THz systems that can perform real-time THz imaging and spectral measurements at ambient temperatures [49]. This is expected to stimulate faster development in compact THz imagers for medical and biological aims, for their effective implementation in security systems for detection of hazardous materials, hidden explosives, and illicit substances.

An alternative approach to THz generation in QCLs at room temperature is the replacement of the conventional THz QCL design with an intra-cavity THz difference-frequency generation scheme under pumping of dual-wavelength mid-IR QCLs [44,50]. The room temperature operation can be achieved as the emission conditions in this scheme do not require population inversion across the THz transition, while mid-IR QCLs can reliably operate at room temperature with high powers. These devices have experienced dramatic progress—their spectral tunability falls within 2.6–4.2 THz making them attractive for THz spectroscopy and multispectral imaging [51]. The peak powers, for instance, at 3.6 THz can be scaled up to 1.4 mW using the distributed-feedback waveguide with Čerenkov phase-matching scheme [52]. THz QCLs processed into double-metal waveguides with surface-grating outcouplers allow to shift the red side of the emission below 2 THz and reach rather effective mid-infrared-to-terahertz conversion with peak power output over 110 µW at 1.9 THz [53].

*Current challenges and possible future trends*: One of the main current challenges in the development of the conventional THz QCLs is room-temperature operation at reasonable power levels. The emitted power at 240 K amounts to 20 mW [49], while the highest operation temperature is −23 ${}^{{}^{\circ}}$C (250 K). Another important challenge for THz imaging applications is a requirement of spatial and spectral quality of the emitted radiation. As THz QCLs are similar to microwave microstrip transmission lines with active region placed between two confining metal layers (waveguides), it results in a highly non-directional far-field beam pattern. To engineer better quality of THz QCL spatial emission profile, specific distributed feedback design of a double-metal waveguide can be used [54]. Single spectral mode and a diffraction limited single-lobed beam can be realized via longitudinal coupling of metallic microcavities through the traveling plasmon waves [47]. In vertical-cavity surface-emitting QCLs, single mode lasing regime, together with its tunability, can be implemented by employing broadband amplifying metasurfaces of sub-wavelength thickness, which allow lasing on low-order Fabry–Pérot cavity modes with good beam quality and high output power at 77 K [55]. The breakthrough challenge remains to implement this approach into room (or close to room) temperature operating THz QCLs.

Finally, it is worth noting the spectral tunability of these devices. As it was already mentioned, room temperature THz difference-frequency generation scheme QCLs operates slightly below 2 THz [53], while conventional THz QCLs design at the highest reported temperature—250 K at 4 THz [49]. The issue to shift to lower frequencies the red wing in emission still remains unsolved and challenging; thus, probable alternative to cover subTHz frequencies can be electronic devices, like Schottky barrier-based frequency multipliers [56,57], CMOS technology-based oscillators [58,59,60,61,62], heterojunction bipolar transistors [63,64,65], or semiconductor superlattices [66,67,68].

### 2.3. High Electron Mobility Transistor-Based Sources

As mentioned above, routes in development of compact room temperature THz radiation sources in subTHz frequencies can be electrically-pumped oscillators or multipliers. Pioneering works [69,70] dedicated to study plasma waves-based emitters revealed a promise in this approach. Further works investigated features of the mechanism behind the emission in more detail [71,72]. Concerning their application, the absolute power of THz emission at room temperature, for instance, in GaN/AlGaN nanometric FETs, was found to be relatively weak, up to 1.8 µW value [73].

*Recent advances:* During the last decade, technologies for fabrication of High Electron Mobility Transistors (HEMTs) or Heterojunction FETs (HFETs), have been developed to the level which allows for using them as the key element in Monolithic Microwave Integrated Circuits (MMIC) which are currently breaching the 1 THz limit [74]. These technologies employ different types of heterojunctions of semiconductors, and the highest operation frequencies $f_{max}$ are achieved for InP-based HEMTs with InGaAs/InAs composite channel and sub-100 nm short gate lengths [75]. Since the first report of extrapolated $f_{max}$ of InGaAs/InAlAs/InP HEMT with 35 nm gate exceeding the 1 THz limit [76], similar parameters were achieved for a 20 nm metamorphic HEMT technology [77] and the $f_{max}$ of 1.3 THz for an extended drain-side recess structure in 75 nm gate InAlAs/InGaAs HEMTs [78]. Although currently available just in a few places worldwide, such technologies are starting to be named as Terahertz Monolithic Integrated Circuits (TMICs) and, by now, have proven to be able to produce efficient frequency multipliers for 500 GHz [79] or 670 GHz [80], mixers for 600 GHz [81], low noise amplifiers for 600 GHz band [82,83,84], and transmitters and receivers for 850 GHz [85] with the record report of achieved amplification up to 8 dB at 1 THz [86].

*Current challenges and possible future trends*: The performance of TMICs as receivers or active multipliers is just starting to approach those achievable with Schottky diodes [87]. Nevertheless, compared to the latter, the TMICs have potential advantages with respect to their functionality and compactness. Furthermore, the development of III-V -based MMICs and TMICs is commonly envisaged as the closest alternative for the Si CMOS technology for digital circuits in order to ensure the continuation of the increasing speed of consumer electronics [88]. However, up to now, the TMICs remain as the proprietary technology which is available for very limited range or users.

### 2.4. Silicon Nanotransistor-Based Sources

*Recent advances:* The performance limits of analog high-frequency electronics-based on the industrial mainstream semiconductor fabrication technologies, i.e., a complimentary metal oxide semiconductor (CMOS) and bipolar CMOS (BiCMOS), are now almost comparable to benchmark setting III/V devices. Field-effect transistors (FETs) and, in particular, silicon–germanium (SiGe) heterojunction bipolar transistors (HBTs), now report cut-off frequencies $f_{T}$ and maximum oscillation frequencies $f_{max}$ exceeding 300 GHz, therefore, directly entering into the THz frequency range [62,89].

Profiting from the technological maturity which ensures a high yield and the ability to construct circuits involving a large number of devices, a variety of different approaches for CMOS-based sources have been proposed. One of the first proposed methods to overcome cut-off frequency limitations used the summation technique of several phase-shifted oscillators, or so-called linear superposition technique, which resulted in −46 dBm gain at 324 GHz [59]. One of the first reports to implement a signal source utilizing the device nonlinearity for efficient generation of signal at higher harmonics resulted in −49 dBm at 410 GHz [58,90]. The latter concept is based on a strong oscillator at frequencies which are typically slightly below or close to the half of $f_{max}$, and the concomitant efficient extraction of from the electrical nonlinearity originating the harmonics. Furthermore, the implementation of so-called triple-push configuration is seen as advantageous over a more standard two-transistor-based push-push oscillator topology in the some aspects, like it allows achieving fundamental oscillations near $f_{max}$ and higher voltage swing. The implementation of this concept, based on the on-wafer measurements resulted in generation of −7.9 dBm at 482 GHz frequency [91]. Yet another efficient method for increasing power by coupling a number of oscillators was reported to generate a −1.2 dBm at 290 GHz and −3.3 dBm and 320 GHz [92].

A standard development strategy of high-frequency circuits, which starts from the on-wafer characterization, can be continued to either waveguide-integrated devices or towards monolithic integration of antennas. One of the first reports on efficient realization of antenna-integrated sub-THz oscillator demonstrated up to −4.1 dBm at 288 GHz [93] emitted into free space.

The strategy that the oscillator drives integrated antenna is almost naturally fits within so-called Colpitts oscillator implementations. The first implementations using a 90 nm CMOS technology reported up to −8.8 dBm at 217 GHz frequency [94]; however, implementations using 65 nm technology and the optimization of the circuit made it possible to reach up to −2.7 dBm at 293 GHz [95], which currently achieves the highest frequency for CMOS-based oscillators of 615 GHz with a −17 dBm of total emitted power [96]. Furthermore, with the help of external synchronization, an array of 30 equivalent densely packed sources were reported to produce up to +9 dBm at 280 GHz [97].

Recent developments in bipolar CMOS technologies, such as 130-nm SiGe with the maximum oscillation frequency reaching 500 GHz, allowed utilization of oscillator concepts and reporting 1 dBm from a single antenna at 245 GHz [98] or −6.3 dBm at 430 GHz [99]. The application of power combining technique on a 64-pixel array resulted to 9.2 dBm radiated power at 420 GHz [100]. A 1 THz barrier was recently breached by coupling the output of 42 oscillators, and the efficient fourth harmonic extraction allowed reporting of a total emitted power of −10.9 dBm at 1.01 THz [101].

Besides the possibility to form clusters of oscillators to increase power, the array of synchronized sources also allows for beam steering. Steering angles of 45 and 50 degrees along two axes were reported in a circuit operating at 338 GHz [102]; it permits a new spectroscopic THz instrumentation based on a set of circuits with different functionalities, like a 550 GHz frequency synthesizer [103].

The classical concepts of nonlinear conversion include approaches utilizing varactor-based solutions as either active circuits [104,105], or fully passive [106,107,108] devices. Although, both nonlinear oscillator and varactor-based approaches produce comparable levels of output power below 600 GHz, the separation of the nonlinear element allows for more efficient nonlinear element, enabling extension of the operation frequencies up to 1.4 THz [109].

*Current challenges and possible future trends:* The ongoing improvement in the performance of FETs is driven almost hand-in-hand with requirements to increase the efficiency in digital systems. This implies the reduction in the minimum feature size, as well as involves innovations, such as introduction of high-k dielectrics, implementing silicon-on-insulator (SOI) structures, exploiting strained active regions, etc. From the progress in the field of analog CMOS integrated circuits, there are indications that CMOS FETs already have approached their speed limits at about 45 nm and 65 nm nodes. However, a theoretical analysis of SiGe HBTs indicates that operating frequencies of 1 THz and beyond are plausible in the future [110].

It must be noted that the progress in the development of active circuits, to a large extent, relies on the availability of high-frequency models for devices. Such models are typically validated for the GHz frequency range, yet their application to the THz range becomes disputable due to the importance to account for a high-frequency transport-related phenomena, such as skin currents or plasma waves excitation, which gain importance at higher frequencies and directly affect the efficiency of mixing [111]. However, since the limits of operation of active part are predetermined by unity gain frequencies which, for CMOS technologies, lay below 300 GHz, even currently existing models, especially those which include distributed nature of devices, also allow creation of successful designs for the extended frequency range.

The main advantage of silicon technologies, regarding their application in THz frequency range, comes from possibilities to form large collaborative acting clusters, allowing the implementation of different than currently established technological approaches or even to enable a new functionality for applications. As an example, one can serve combination of electronic and photonic concepts to extend facilities that other technologies cannot provide currently.

### 2.5. Resonant Tunneling Diodes

Resonant tunneling diodes (RTDs) consist of two or three ultrathin and closely spaced layers of a large-bandgap semiconductor embedded within a lower-bandgap semiconductor (double- or triple-barrier RTDs). They burst onto the scene as room-temperature sources of THz radiation, when a team at the Massachusetts Institute of Technology (MIT) Lincoln Laboratory reported THz emission up to 420 GHz in 1989 [112] and, later, in 1991, even up to 712 GHz [113]. RTDs were then also explored at the University of California at Santa Barbara (UCSB) as gain elements in pulse-forming nonlinear transmission lines [114]. However, progress regarding high-frequency operation, and especially the enhancement of the output power, quickly stagnated, so the hope for a new emitter option for practical applications gradually vanished [115]. The hope was revived when, around 2010, two teams reported nearly simultaneously to have reached and surpassed the 1.0-THz milestone of the fundamental oscillation frequency [116,117]. This advance built, on one hand, on an improved growth technology of the semiconductor heterostructures, which allowed for thinner barriers. However, it also reflected a better understanding of the electron transport through the RTD, especially regarding the role of Coulomb interactions [118]. The maximal fundamental operation frequency could now quickly be increased [119], to reach a record value of 1.98 THz in 2017 [120,121].

*Recent advances:* The status of the research of RTD devices is described in two informative recent review articles [115,122]. While RTDs are explored as radiation sources for applications in imaging [123], it is clear that limitations arise because of the limited output power. Peak values are on a sub-mW level at 0.3 THz to roll-off to tens of µW at 1.0 THz [115]. This is a consequence of the sub-micron size of the RTDs arising from the need for a low device capacitance as a price for the high operation frequency. Recently, incorporation of RTDs in transmission lines has allowed for a larger diode footprints and a higher output power, reaching 1 mW at 260 GHz [124]. Another approach for power enhancement is the use of power combining arrays [122]. A record of 89 RTD elements was combined monolithically in an array. This led to a total emitted power of 0.73 mW at 1.0 THz [125]. The power scaled linearly with the number of elements, and frequency locking was not observed, indicative for incoherent (non-superradiant) coupling.

*Current challenges and possible future trends:* The quest for higher power certainly will remain to be a dominant motivation for research in the years to come. Additionally, the continued exploitation of the inherently nonlinear response of the devices may lead to a further increase of the fundamental operation frequency and to the development of novel multipliers, mixers and other nonlinear devices [126]. With regard to the nonlinearity, triple-barrier RTDs may again find increased attention. The use of three tunneling barriers allows for more flexibility in the shaping of the current-voltage curve [115]. This was already exploited in the realization of oscillators, leading to the demonstration of fundamental-frequency operation at 517 GHz in 2010 [127]. But, because the current density is lower than in double-barrier RTDs, the output power of the oscillators is lower, which dampened the interest in continuing the investigation of such sources. However, they have now been found to be attractive as THz detectors for room-temperature operation. Properly designed, they exhibit a strong nonlinearity of the current-voltage characteristics at the onset of the resonance. This nonlinear dependence is more pronounced than it would be if determined only by the temperature. The reason for the steeper rise of the current versus voltage is the alignment of the subbands of the adjacent quantum wells [115]. This allows for a high responsivity (i.e., ratio of the electrical output per optical input), exceeding the thermal limit. Recently, an impressive voltage responsivity of 6.6 × $10^{4}$ V/W at 260 GHz and ambient temperature has been reported for a detector fabricated in the InGaAlAs material system [128]. Although the noise equivalent power (NEP) (i.e., minimum optical power required an output signal-to-noise ratio equal to 1 in one hertz bandwidth; details can be found, for instance, in Ref. [129]) value has not been specified, the fact that the device can be operated in a zero-bias mode is promising with regard to low-noise properties.

Regarding future trends, the question arises as to the potential of van-der-Waals materials as a new basis for RTDs (or other diode-type emitters to be fabricated with them). Theoretical studies suggest that oscillators based on a stack of graphene/hexagonal boron nitride/graphene could operate at a fundamental oscillation frequency of hundreds of GHz at similar output power as existing RTDs [130]. Other simulation work even predicts emission at several THz if classical RTDs are coupled to graphene plasmons in a transmission line arrangement [131]. While RTDs and other devices with negative differential resistance are being fabricated with van der Waals materials [132,133], we are not aware of any reports about experimental studies of THz emitters based on such devices.

### 2.6. Vacuum Electronics

As imaging applications usually benefit from high radiation power, vacuum electronic devices, which can provide exceptionally high output power, principally are of high interest as radiation sources [134]. However, one does not find them to play a central role in THz imaging applications until now. This has several reasons, and different ones for the different types of devices. This can be their size (gyrotrons, free-electron lasers), the need for high magnetic fields (gyrotrons), frequency limits (klystrons), or limited lifetimes and unreliable supply of components of the devices (traveling-wave tubes), to name some often-stated factors. With regard to compactness, output power and coverage of interesting frequencies, the backward-wave oscillators and amplifiers developed by Teraphysics Corp., USA, appear highly attractive [134]. They have the footprint of a credit card [135,136] but achieve high output power by their use of sophisticated micromaching with diamond used in critical parts of the devices [137]. The company built emitters up to 650 GHz [137] but seems to focus on wireless communications at lower frequencies more recently [135]. We are not aware that devices are available to users on the open market.

## 3. THz Room Temperature Detectors and Arrays

Compact room temperature THz imaging systems and their usage in real working conditions define a large variety of specific requirements for sensing devices. On the one hand, they should be sensitive, exhibit broadband operation and high dynamic range; on the other one, devices should be reliable, electrically and mechanically stable, and, which is of a particular importance, have a possibility to be combined into detector arrays. This criterion imposes a strong preference for planar-technology-based solutions. There are sensors cameras already commercially available for THz imaging [138]; however, here, we will restrict our attention only to several types of devices, which only recently experienced a rapid evolution.

### 3.1. Field Effect Transistor-Based Detectors

**Figure 2:**
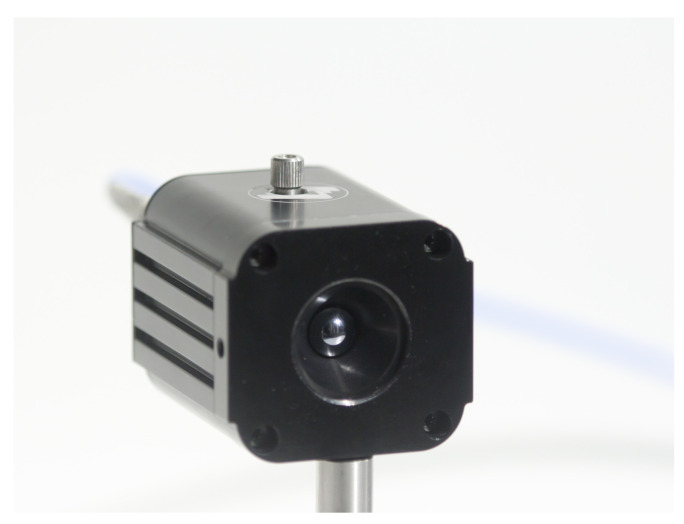
Photo of silicon lens coupled FET-based THz detector equipped with low-noise amplifier. Courtesy of MB “Terahertz Technologies”, Vilnius, Lithuania.

*Recent advances*: First concept-breaking reports that the field-effect transistors can be utilized as THz detectors (Figure 2) were performed on devices without any dedicated antennas [139,140,141,142]. This research was mainly inspired by the predictions of Dyakonov and Shur [143] regarding the peculiarities of propagation of plasma waves excited in two-dimensional electron gas. The first antenna-coupled devices, fabricated using 65-nm CMOS technology and designed for 600 GHz frequency range, reported moderate sensitivity in NEP terms of 300 pW/$\sqrt{\mathrm{Hz}}$ [144]. However, improvements in detector design, as well as implementation of detectors in smaller technological nodes, such as 90-nm or 65-nm, resulted in the performance change that is now approaching to the NEP value of 10 pW/$\sqrt{\mathrm{Hz}}$ [145,146,147]. The exceptional maturity of silicon technologies allow integration of antennas with resonances going deep into the THz frequency range [148,149].

Moreover, detectors with substrate lens demonstrate broadband operation with nearly flat frequency response from 400 GHz up to 1.5 THz [150] and can be used with THz radiation generated employing low-average-power sources, like photomixers [151] or photoswitches [152].

Furthermore, advantages of silicon technologies bring the ability to form large detector arrays which can be used for imaging applications [153,154,155,156]. Moreover, the availability of both types of components, i.e., sources and detectors, makes it possible to realize all-electronic silicon-based applications spanning from raster-scan imaging [146,157] to near field imaging [158]. It is worth mentioning a very recent study [159] dedicated to the sensitivity issues of nanometric field effect transistors as THz sensors.

*Current challenges and possible future trends*: Since first demonstration of FETs in THz imaging [160,161], the utilization of FET-based detectors for the THz frequency range is still evolving. The current success, as well as further improvements of performance, are strongly related to the general development in the field of high-frequency integrated circuits and correlate with the maturity of technology. Furthermore, the introduction of novel two-dimensional materials, such as graphene, allows involving additional mechanisms, such as carrier heating [162,163,164], ballistic transport [165], or tunneling currents [166], which might be exploited to enhance rectification properties.

### 3.2. THz Diodes-Based Sensing and Microbolometers in THz Imaging

*Recent advances*: As important other members in the family of room temperature THz sensors, one can mention diode structure-based devices and microbolometers [167]. As a classical example of diodes, one can underline Schottky diodes, which have already long been and continue to be one of the most useful THz devices both in detection and THz emission schemes as high as 3 THz frequencies [87]. The identification of CMOS technology as a promising platform for the development of THz devices [168] suggested different ways to make THz chips, as well as Schottky diodes [169], enabling reliable and circuits-friendly solutions. Concerning materials choice, GaAs [170] and GaN-based structures [171] remain as the main platforms for sensor development. These materials successfully served for the fabrication of nanodiodes for THz detection: GaAs-based devices, for instance, were able to reach 1.5 THz with 300 V/W sensitivity at room temperature [172], while equivalent values in operational bandwidth and sensitivity values for GaN nanodiodes were found to be of 200 GHz and 50 V/W, respectively [173,174].

The so-called bow-tie diodes operating on non-uniform carrier heating effects demonstrate broader operational bandwidth reaching 2.5 THz for all investigated materials GaAs [175], GaAs/AlGaAs [176], and InGaAs [177], with the highest and nearly independent of frequency sensitivity of about 10 V/W below 1 THz for InGaAs sensors. They were found to be well-suited for multispectral THz imaging aims [178,179], and, together with their reliability and fast response times below 7 ns [180], can be implemented in real imaging systems to discriminate even weakly absorbing objects when tuned to heterodyne [181] or homodyne [182] operational modes. Recent technological innovation making use of new growth conditions [183] enabled increase of the sensitivity of 13 V/W and reduction of the NEP below 1 nW/$\sqrt{Hz}$ at 0.6 THz due to strong built-in electric field effects.

**Figure 3:**
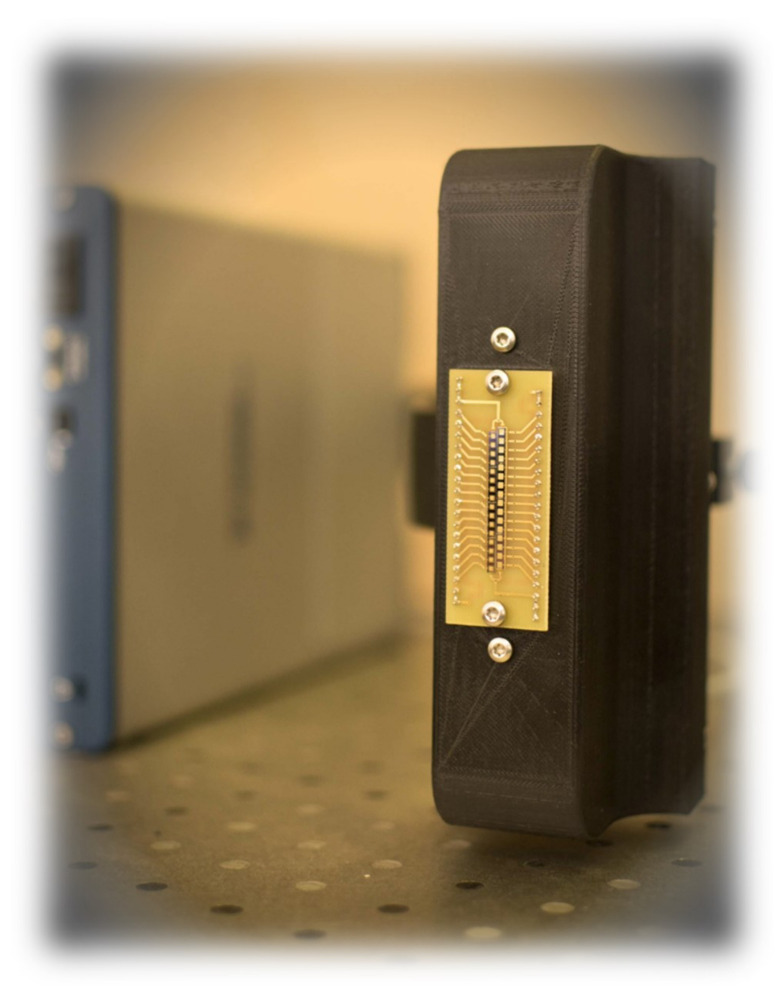
Photo of titanium-based microbolometer THz camera. It is composed of a linear array consisting of 2 lines with 16 pixels each. The left line is used for the detection of 700 GHz, and the right one for the detection of 300 GHz. It was realized via coupling of the sensors with antennas of relevant designs. The titanium bridge of 12 µm and 2 µm in length and width, respectively, was electrodeposited on the silicon nitride (SiN) membrane of 2-µm thick. The membrane was etched to reduce the heat capacitance and to increase the speed of operation. More details can be found in Refs. [188,190]. Courtesy of Laboratory for Microelectronics of Faculty of Electrical Engineering at University of Ljubljana and Luvitera Ltd., Vilnius, Lithuania. Photo: courtesy of Linas Minkevičius.

Microbolometers, as a rule, suffer from a relatively long response times and pose an obstacle to implement them into real-time THz imaging systems. However, renaissance of microbolometers can be associated with their elegant demonstration in real-time THz image experiments using a commercially available infrared uncooled, vanadium oxide-based microbolometer focal-plane array camera under illumination of THz QCLs [184,185]. Later, amorphous silicon bolometers for real-time video rate 2D imaging were also fabricated monolithically above a CMOS read-out electronics delivering 25 images per second [186,187]. High quality images of broadband THz beams covering the 0.1–2 THz range were recorded while maintaining SNR of 10 for detected THz power as low as 25 nW. High sensitivity values have reached MV/W; therefore, these devices can be found well-suited for detection of very low emission sources, like femtosecond laser-based THz emitters. Ti-microbolometers (Figure 3) exhibiting room temperature sensitivity values of 200 kV/W, NEP less than 20 pW/$\sqrt{Hz}$, and the response time in the 5 µs range are also sensitive and fast enough to precisely monitor both coherent THz emitters or spatial profiles of frequency-domain spectrometers [188,189]. Because of their high sensitivity, these devices can successfully be applied for medical aims, for instance, to identify carcinoma tissues [190].

Novel type of microbolometers, including microelectromechanical systems (MEMS), seem to be promising to tackle the problem of slow response time [191]. GaAs was found to be well-suitable material for these devices: Using a doubly clamped GaAs MEMS beam resonator as an ultrahigh sensitive thermistor, the bolometer achieved not only high sensitivity but also an operation bandwidth of several kHz, which is more than 100 times faster than other uncooled THz thermal sensors. The obtained NEP was found to be in the range of 90 pW/$\sqrt{Hz}$, while sensitivity was 3300 V/W [192]. These parameters enabled the employment of bolometers of this type for the rapid scan with a stage speed of 25 mm/s with no degradation in the resolution [193].

*Current challenges and possible future trends*: Diode-based sensor further development is mainly aimed at finding planar solutions for sensing arrays and an increase in sensitivity via internal electric field engineering, as, for example, in the case of bow-tie diodes composed of InGaAs or GaN/AlGaN materials.

Concerning microbolometers, microelectromechanical systems (MEMS) technology shows a promising development trend. They were realized in silicon wafers, where the readout integrated circuits had been prefabricated to employ VOx film as a bolometer active layer, whose changes in the resistance can be detected and converted to the output voltage or current signals [194]. Other routes in microbolometers evolution are related to search of new materials, for instance, $La_{0.7}Sr_{0.3}Mn_{O3}$ thin films as active device layers [195], silicon-on-insulator (SOI), CMOS technology employing different Si-based temperature sensors, such as metal-oxide-semiconductor field-effect transistor, pn-junction diode, and various resistors as possible antenna-coupled bolometers [196]. An interesting concept can be their confluence with metamaterials, e.g., metallic split-ring resonators, integrated into micro-bridge structures of THz microbolometer arrays to enhance the absorption of THz radiation in different materials, like thermal sensitive vanadium oxide film [197]. As a THz absorption layer, one can use specially designed metamaterials with variation of the electrical resistance in polydimethylsiloxane mixed with black carbon thermosensitive composites [198].

Special attention is required for metamaterials. Since their first demonstration in THz frequencies [199], metamaterials proved themselves to be a useful and convenient solution in designing passive and active THz components [200,201] that can successfully be used in THz imaging systems.

Since metamaterials can exhibit perfect absorption properties [202,203], their combination with 2D materials, like graphene [204], can be a promising route for the evolution of modern THz microbolometers.

## 4. Diffractive Optical Components and Beamforming (Beam Engineering) in THz Imaging

Convenience of room temperature THz imaging systems in regard to their dimensions and on-chip solutions remain a priority to achieve their wider technological adoption. As a rule, the development of solid-state-based solutions for miniaturization of the imaging system active components—emitters and detectors—attracts more attention than passive optical components, like lenses, mirrors, beam splitters, etc., despite the fact that these can also be bulky and, in a majority of cases, require precise optical alignment.

Next, we will discuss some routes in the development of diffractive optics elements. It aims to demonstrate that such elements can provide a platform to fabricate optical components for the needed wavelength range and to manage THz beam profile via interference effects.

**Figure 4:**
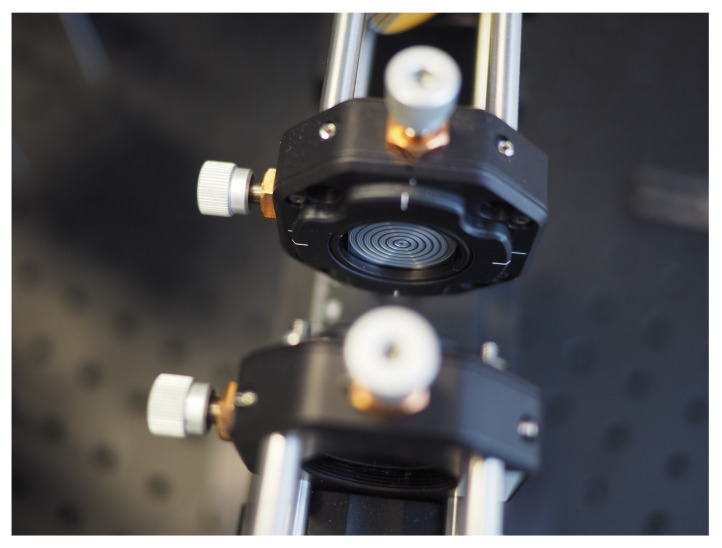
Photo of high-resistivity silicon-based Bessel zone plate placed in the optical mount. The diffractive optical element is prepared by the laser ablation technology. More details on fabrication technology and the zone plate design can be found in Ref. [213]. Photo: courtesy of Domas Jokubauskis.

*Recent advances*: Extending previous very recent comprehensive overviews on THz diffraction elements [205,206] dedicated to optically passive elements, like gratings, diffractive lenses or their arrays, diffractive components on spatial terahertz modulators, or holograms, one also needs to emphasize novel structures for beamforming (beam engineering) [207]. Beam management can be arranged in a relatively cheap and flexible way relying on optical graphite features [208], printed passive beam guiding or antireflective optical elements [209,210]. To reduce the losses, high-density polyethylene (HDPE) materials or polyamide layers can be replace by high-resistivity silicon-based multilevel phase Fresnel lenses [211] or zone plates (Figure 4) fabricated, for instance, by laser ablation technology [212]. It was shown that such components can successfully be used for THz beam focusing, as well as beam-engineering, for instance, in Bessel or Fibonacci-beam generation [213,214]. The approach works well even for large apertures, up to 50 mm [215], and it allows for reaching nearly 90% transmittance of silicon antireflection layers and produce focusing by binary zone plates [216].

The THz beam engineering, as mentioned above, can also be realized using metamaterials [217,218]. THz beamforming and its manipulation via amplitude, phase, and polarization can be achieved using metasurfaces, uniquely engineered thin surfaces with two-dimensional specifically shaped arrays of sub-wavelength-spaced and sub-wavelength-sized resonators. They can be found as useful instruments, e.g., for a THz vortex-beam-generation [219] and a phototunable effective (reaching value of 99.93%) transmission THz intensity modulation via so-called dielectric Huygens’ metasurfaces [220]. Metasurfaces provide a large variety of opportunities to design novel components for THz polarization conversion [221] as conventional THz wave plates made of natural birefringent materials typically suffer from a low efficiency, narrow bandwidth, and substantial thickness. As an another beautiful example, polarization-controlled THz superfocusing can be realized by emitting high-efficiency radially convergent surface plasmon polaritons into free space to form a focal spot beyond the diffraction limit [222].

*Current challenges and possible future trends*: Reduction of dimensions in THz imaging systems is one of the main persistent aims and where metamaterials and metasurfaces can contribute as important solutions. In particular, the combination of sub-wavelength optics and plasmonics is expected to grow in the upcoming years [223]. Deserving of mention is single-pixel near-field imaging, which enables spatially resolved sub-wavelength patterns while recording the correlated intensity on a single-element detector [224], as well as the evolution of incoherent imaging using photonic-integrated devices—optical phased arrays [225]. One can predict the rise of metasurface uses in THz pulse shaping or in novel dispersion-engineering tools [226] as it was done already in flat optics components at visible light [227].

## 5. Spatial Filtering Methods in THz Imaging

Spatial filtering represents well-known techniques in infrared and visible light microscopy which are used to investigate low absorbing objects, as commonly found in biology. Conventional or bright field imaging is usually employed if samples experience strong absorption; dark-field imaging provides good contrast for weakly absorbing object, since it only captures high-angle scattered light. Phase contrast is used for unstained and transparent biological samples, allowing visualization of their shape and density variations [228]. Pioneering work in THz range was dedicated to investigate dark-field imaging aiming to enhance the obtained image contrast through the analysis of scattering and diffraction signatures [229].

*Recent advances*: Very recently, the spatial filtering concept was extended to THz by application of two spatial filtering methods—phase contrast and dark-field—to resolve weakly absorbing objects in THz imaging at monochromatic light of 0.3 THz frequency [230]. It was shown that both phase-contrast and dark-field methods in THz range can exhibit enhancement in images contrast up to 10 dB and 30 dB, respectively, and an order of magnitude increased SNR, thus opening a promising route for functional applications. Apart from the amplitude and phase, use of the polarization states in spatial filtering experiments can offer additional advantages and possibilities. It was demonstrated in visible light that an S-waveplate that can convert a linearly polarized light into radially or azimuthally polarized light, and it can also be used for isotropic edge enhancement. Unlike the standard amplitude and phase-based Fourier filters, which are independent of the state of polarization of the illuminating beam, the S-waveplate indicates the state of polarization [231].

*Current challenges and possible future trends*: These developments will impact THz uses in medicine and biological applications, where weak absorbance of THz radiation, in many cases, is an inherent feature of investigated objects, and where it is a particular interest to differentiate water-induced absorption from the tissue effects. The latter issue requires polarization-sensitive filtering; therefore, one can expect further development in this field. It is worth noting initiatives to apply spatial filtering in pulse shaping to monitor and control emission of compact THz sources. One can mention, for instance, THz QCLs by design of two back-to-back mounted elliptical silicon lenses and an opening aperture defined on a thin gold layer between the lenses [232] which enables filtering of the non-Gaussian beam from the QCL to a nearly fundamental Gaussian beam. As for multispectral THz imaging utilizing broadband THz pulses, an important innovation can stem from Fresnel apertures and filters that provide possibilities not only for the pulse shaping but also generation of longitudinal THz fields [233], hence exciting relevant applications.

## 6. On-Chip Solutions in THz Imaging

As already mentioned above, imaging systems operating in real operational conditions must be compact, reliable, and convenient to use. Technically, it means presence of minimal optical alignment issues, enhanced functionalities, and essentially reduced power consumption. CMOS technology can serve as a suitable platform to integrate on-chip emitting and sensing components of THz systems [62]. However, the most bulky elements in THz imaging systems, as a rule, are passive optical components, such as focusing mirrors or beam splitters. Therefore, the development of THz integrated electronic and hybrid electronic–photonic systems can stimulate advances delivering important functionalities for applications in sensing and imaging [234]. In what follows, we discuss how routes in the development of on-chip solutions for passive diffractive optics-based elements can be used in THz imaging.

*Recent advances*: Silicon lenses can be useful tool to collect the incident THz light, for instance, into antenna-coupled AlGaN/GaN based HEMT detectors: Silicon hyperspherical lens helps to concentrate even incoherent THz light on a detector chip assembled on the planar surface [235]. In case of employment in THz imaging of focal plane sensors arrays, 3D printed arrays can be used. They are diffractive multi-zone lenses, offering both good efficiency and needed uniformity, and thus enabling improvement in the SNR of THz HEMTs based detectors by more than one order of magnitude [236]. Moreover, the proposed technology provides a route to produce cost-effective, reproducible, flat optics for large-size THz HEMTs—detector focal plane arrays.

Zone plates can be attractive ingredient in the miniaturization of the THz image recording setups as their rely on planar technology solutions. As an illustrative example, they can serve their on-chip integration with InGaAs-based bow-tie THz detectors [237]. The top surface was used to process the InGaAs detector array, whereas the bottom metalized surface of the InP substrate was structured to provide the focusing zone plate elements. Such an integration in a single chip allowed one to increase the signal by at least an order of magnitude.

As an another example of planar solution that deserves notice is the standard 180 nm CMOS technology-based diode microbolometer sensor which is monolithically integrated with a broadband THz metamaterial absorber [238]. The given approach allowed demonstration of uncooled low-cost THz imager capable of being used in a stand-off imaging applications.

Planar solutions of integration can be realized using special kind of waveguides. One can note, e.g., comb-shaped waveguide based on the excitation of coupled spoof surface plasmon mode can be found to have a pronounced effect for the enhancement of fingerprint detection sensitivity in the THz range, and it can be an essential tool in biomedical imaging [239].

*Current challenges and possible future trends*: It is rational to suppose that the trend of on-chip components integration will be kept. Integrated circuits based on CMOS technology are expected to be dominated in the development of subTHz and THz range imaging systems because of their advantage in design and fabrication of compact, low-cost, and low-power consuming microelectronic circuits. Graphene–CMOS integration can evolve as one possible technological trend [240]; a significant breakthrough can be predicted under use of plasmonic interferometry based on graphene field-effect transistors with special antennas, thus providing precise measurement of phase difference between two arbitrary phase-shifted THz signals [241].

## 7. THz Computational Imaging

Computational imaging (CI) presents a highly integrated platform, enabling a tight link between modern sensing devices, advanced optics and post-recorded signal processing [242]. CI allows for “correcting” imperfect physical measurements made by sensing system, therefore improving facilities of the image recording hardware in identification or discrimination of objects under test. In particular, when measurements are noisy, objects are placed in scattered package or super-resolution is needed, deep learning approach can be found as especially attractive tool [243]. Therefore, CI principles can be used to construct an instrument which, compared to a conventional image approach, can provide higher image quality and desired capability, together with reduced dimensions, used power, and cost. It was shown that CI using THz radiation can strongly extend boundaries of application in medicine, for instance, in studying human skin [244]. Computational sampling methods, which combine computational algorithms with optical imaging techniques, can improve the sampling speed and image quality, it can determine spatial distribution of THz fields with the sub-wavelength accuracy via probe-beam encoding [245]. To realize a fast “colored” imaging system, digital light projector was used to illuminate the object with different light patterns and correlate them via measured by three spectrally-filtered single-pixel photodetectors to produce a full-color high-quality image [246]. In THz range, hyperspectral THz imaging can be realized via nonlinear THz generation with time-resolved field measurements in time domain spectroscopy techniques [247]. Experimentally, it was demonstrated via time-resolved nonlinear ghost imaging, a technique based on near-field, optical-to-terahertz nonlinear conversion and detection of illumination patterns [248].

*Recent advances*: **THz compressed sensing:** Solutions in THz range display some specifics due to lack of room temperature high power sources required in their employment together with recording focal plane arrays. To overcome the problem, the compressed imaging using single pixel camera can be used instead [249]. Compressed imaging was originally proposed to reconstruct an image using low-dimensional detection and then employing computational algorithms to solve the ill-posed inversion [250]. The compressing approach in THz can be demonstrated using optical photoexcitation of semiconductors to dynamically and spatially control electromagnetic properties of a semiconductor mask to collectively form a THz spatial light modulator [251,252,253]. Another type of pixel-controlled tunable mask is metamaterial patterns, including the formate of plastic plate coppered with designed distributions [254], spinning disk plated with metal diagrams [255,256], and all-electric controlled active metamaterial real-time spatial mask [257]. By using the optical induced modulation, a THz video (32 × 32 pixels by using pulse radiation) with the frame rate of 6 Hz of has been achieved [252].

**THz holography:** Holograms, in addition to the light intensity, records phase changed by illuminated objects, hence allowing precise mapping across the imaged object if it is low absorbing or event transparent in THz range. Moreover, it provides a possibility to reveal pattern of object thicknesses, density distribution and relief features. The employment of highly coherent THz sources allows for achieving high-fidelity amplitude and phase reconstructions, as well as indicates ways of how to reconstruct images of complex dielectric targets [258]. Recent activities are dedicated to increasing quality of THz holography and extending new areas of application in this rapidly evolving field of THz optics. THz pulse time-domain holography, in contrast to that of using monochromatic source, allows high-resolution amplitude and phase THz imaging with mapping spectroscopic information across the imaged object [259]. To realize compact holograms recording schemes, THz QCL can be used, enabling demonstration of real-time digital holography exploiting high spectral purity and the mW output power of the laser combined with the high sensitivity and resolution of a microbolometric array. Moreover, it was shown that phase and amplitude information of whole samples can be recorded in a one-shot exposure [260]. To overcome issue of slow frame-rates and low resolutions, a full video-rate THz digital holography system operating at 2.52 THz was developed, 2D digital reconstructions of samples were performed at frame-rates of 50 Hz, and images of samples concealed in common packaging types were recorded, illustrating its suitability for non-destructive applications [261].

**Figure 5:**
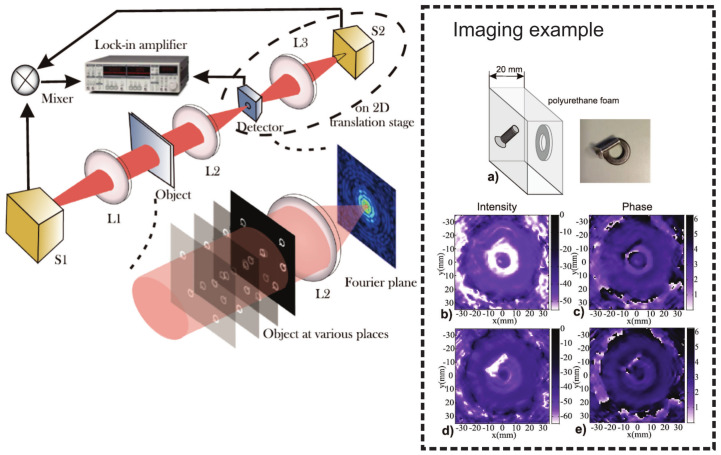
Schematic of the Fourier imaging based on the heterodyne detection of nanometric FET detector at 300 GHz. Left inset: Sketch of the imaging setup. The scene is illuminated by radiation from source S1. The spatial Fourier spectrum of the scene is written in the focal plane of lens L2 (as indicated by the schematic on the lower right side of the figure) and recorded with the raster-scanned single-pixel detector. S2 works as local oscillator and is focused onto the front side of the detector by L3. L3 and S2 share the translation stage with the detector and are moved together with it (as symbolized by the dashed ellipse). Right inset: 3D imaging example (**a**) Left side: Washer-and-screw scene. The THz beam impinges from the left and hits first the screw and then the washer. Right side: The photograph of the washer and screw placed on a table with approximately correct projectional view. Middle panels: Intensity (**b**) and phase images (**c**) for a reconstruction distance equal to the position of the washer; lower two panels: Reconstructed intensity (**d**) and phase (**e**), but for the distance equal to that of the screw. Figure modified from Ref. [262].

**THz Fourier imaging:** Very recently, Fourier imaging recording the data in the Fourier domain folding the 3D information into 2D in the focal plane of the imaging system and reconstructing the 3D scenario by expanding the 2D data numerically was developed by Yuan etc. [262]. As delineated in Figure 5, the heterodyne detection at 300 GHz based on the nanometric FET-based sensor is utilized to capture the Fourier spectrum of the imaged target. The competitive low noise level and phase recording capability of the system provides the 3D image possibility with a high dynamic range of 60 dB, large imaging area of 5.5 cm diameter, and good resolution. The latter covers both lateral (on the order of 2 mm, which is close to the theoretical resolution of 1.84 mm and two times better than conventional imaging with the same system configuration) and depth range (2 cm). The strength of Fourier imaging is to recover 3D space information by sequential object-plane reconstruction via the variation of the object distance in the back-transformation process. The washer and screw imaging results show that two opaque targets, although they are in each others beam path, can be distinguished from each other using the field map of a single focal-plane frame. Different from the Fourier imaging realized using monochromatic radiation by Yuan et al., Guerboukha et al. explained the Fourier domain as k-vector space by introducing the pulsed THz illumination [263]. In this technique, the broadband radiation unfolds the Fourier spectrum in the radial dimension, cooperating with a single-pixel detector scanning in the angular direction along a circular path, and the Fourier spectrum is recorded. Due to the coherent origin of THz TDS operation scheme, the recorded Fourier spectrum contains both amplitude and phase information. By adopting a solid mathematical formulation, the hybrid inverse transform can rebuild the complex imaged characteristics.

**THz 3D vision:** Three-dimensional THz imaging becomes more and more promising as the generation and detection technologies have been developed rapidly. Apart from well-established THz tomography technique, intelligent ideas were taken from the optical range to cope with obstacles in different imaging scenarios. Recent achievements for multi-thin layers 3D scheme reconstruction witness this by the works of ‘Shape-from-focus for real-time terahertz 3D imaging’ [264], ‘Multi-layered full-field phase imaging using continuous-wave terahertz ptychography’ [265], and ‘Coherent reconstruction of a textile and a hidden object with terahertz radiation’ [258]. All of the three methods were developed by using the cameras working at THz range to scan in space. The difference is that shape-from-focus technique scans in longitudinal direction and localizes the best focus position in the stack of images for each pixel and then reconstructs the object in 3D due to the short depth of field. Ptychography needs to scan the camera in 2D lateral directions and then rebuild images by processing many coherent scattered interference patterns generated by one constant function moving laterally by a known amount with respect to another constant function.

**THz super-resolution:** Super-resolution is a topic of great concern in the realm of optical imaging, and it also occupies an important position in THz band. Apart from the method to obtain near-field information maintenance in the far-field region, such as the nano-imaging demonstrated in the latter content, a new artifice super-resolution orthogonal deterministic imaging (SODI) is a accomplished by introducing the spacial distributed mask as the “artificial blinking fluorophores” [266]. The introduction of the blinking mask in SODI alleviates the problem of lacking fluorophores at THz frequencies. By controlling the form of the masks (pattern distribution, amplitude or phase), and reconstructing formulation in different orders, a 1.4 mm resolution at 0.32 THz with few frames, which is touching the resolution limitation, can be validated.

**New design:** As a breakthrough in evolution of compact computational THz imaging one can assume development of THz source system-on-a-chip specifically designed for computational imaging with a single-pixel camera. The source consisting of an 8 × 8 array of independent, frequency-unlocked THz sources radiate maximum 10.3-dBm power at 0.42 THz and, together with spatial modulation of 10-MHz rate, can capture transmission mode images at 25 frames/s with 40-dB voltage dynamic range [100].

*Current challenges and possible future trends*: As a rule, in real implementation conditions, for instance, in security or medicine, THz imaging needs to be done through scattering materials. It implies a search of new imaging techniques to tackle these problems [258]. An imaging system using broadband illumination typically faces the challenge of slow time-domain pulse sampling. A substitution is to use asynchronous optical sampling which is usually more expensive. Relevant to this, new techniques, such as special design delay lines with fast scan capability or cheaper bottom-layer equipment upgrade in asynchronous system implementation, can be the development trends. Compressed imaging enables to solve the problem of insufficient pixels of detection unit in THz regime in a certain extent; however, it leads to an extended imaging recording time introduced by mask modulation and demodulation, which, as a consequence, decrease the imaging frame rate. As the development of the detection array, this problem can be solved from the solid state aspect. Furthermore, one can expect focused efforts to resolve these issues, as well as effective involvement of machine learning architectures and deep neural networks, in particular, in effective computational image formation, as has been accomplished in optical range [267].

The drawback of THz holography is that the resolution is constraint or the imaging speed efficiency is low. In a far-field imaging area, the resolution is inverse proportional to the object distance. However, the object distance of off-axis holography setup is not allowed to be shortened to a small number since the reference beam must not interact with the object. Coming to the in-line holography, the imperfect reference beam will induce ghosting as noise, which will lead to a resolution deterioration. The most widely used ways to alleviate the ghost problem are averaging multi-frame of data or recovering the phase by iteration. Both methods are time consuming. The lacking energy at THz frequencies constrained the imaging field of view and target object distance of holography. This makes harsh obstacles for environment practical applications, such as fire scene, rescue after earthquake, battlefield, etc. Alternatively, it can be done without usage of reference beam employing so-called ptychography, a non-holographic solution of the phase estimation problem. In this technique, one can calculate phase relationships between different parts of the scattered illumination with a incident coherent beam [268]. Despite the fact that only intensity is measured, it allows for reaching lateral resolution below 2λ and depth variations as low as λ/30.

When phase information can be found not essential, 3D images can be obtained by already well-known 3D tomography approach [269] based on mathematical procedures of tomographic reconstruction produced from multiple projectional image recordings [270]. It enables not only 3D visualization and dimensional measurements but also accurate assessment of the structure, geometry, and morphology of the object under test.

The other important issue related to the finding of optimal ways to minimize a coherent source and a camera (without lenses) in the set-up as it was considered in THz imaging [271] and extend it to THz holography. One can expect a new breakthrough relying on the polarization multiplicity and rewritability of a computer-generated meta-hologram in THz range to host unique merits for exploring real-time metasurface-based cryptography [272].

## 8. THz Nanoimaging and Nanoscopy

Obviously, the Abbe diffraction limit of focusing is a major limitation of THz imaging with free-space radiation. Early attempts to overcome this restriction were based on ideas derived from SNOM (Scanning near-Field Optical Microscopy), developed in 1981, for the visible and IR spectral regimes, which utilizes metal-coated tapered waveguides, usually glass fibers, whose ends are brought close to the object-under-test for near-field imaging. For the THz and microwave regime, tapered coaxial cables were developed which allowed for sub-wavelength spatial resolution but had low throughput [273]. Another apertureless approach to sub-wavelength resolution is possible with TDS systems which utilize electrooptic detection. If the object-under-test is placed in close proximity to the electrooptic crystal, then one is able to detect the THz radiation passing the object under near-field conditions with a spatial resolution determined by the optical read-out beam (with typically several µm spot diameter) [274,275]. Apertureless sub-wavelength imaging with TDS systems is also possible employing photoconductive THz field detection. A prominent approach uses freely positionable cantilevers with photonductive switches, capable of generation and detection of microwave and THz pulses, integrated on them [276]. These are activated mainly with laser pulses in the 800-nm wavelength range using transferred low-temperature grown GaAs as active material [277]. More recently, probe tips with Be-doped InGaAs/InAlAs photoconductive material for 1550 nm wavelengths have been introduced [278]. Finally, laser THz emission microscopy (LTEM) should be mentioned, which is capable of a spatial resolution below 3 µm [279,280]. The technique is based on THz-pulse emission from surfaces of semiconductor, superconductors, etc., following their excitation by femtosecond laser pulses; the emission is sensitive to surface modifications of chemical or physical character.

While the techniques mentioned above reach a spatial resolution on the micrometer scale, three imaging modalities have broken through to the nanometer regime. These are THz imaging through nanoslits, s-SNOM (scattering-type SNOM), and THz-STM (THz scanning tunneling microscopy), the latter having reached atomic spatial resolution.

*Recent advances*: **THz sensing and imaging through sub-micron slits:** Considering the first approach, near-field sensing and imaging with any type of nano-scale aperture is in principle capable for improved spatial resolution. However, the challenge is to achieve a high enough throughput for a good SNR. This is possible if one accepts electric field confinement in only one direction using micro- and nano-slits in thin metal films, single ones or arrays thereof, with a length which corresponds to a half-wavelength resonance at the targeted THz frequency [281,282]. With special fabrication techniques, the slits can be brought to a width deep in the nanometer and even sub-nanometer regime [283,284,285]. The extreme wave confinement brings forth intriguing phenomena, such as a strongly enhanced absorption cross-section of molecules, conducive to single-molecule detection [286]. Imaging with sub-micron slits (in contrast to local sensing) is not readily practical, as raster scanning of the object relative to the slotted mask must be performed while near-field conditions are preserved. First, examples of image generation, therefore, proceed differently: A mask with a dense array of slits is attached in a fixed way onto the specimen, while not every point of the specimen is, thus, accessible to the radiation and the limited regions, which experience beneficial contrast changes and enhanced spatial resolution (in one direction) [287].

**THz s-SNOM:** More readily suited to raster-scan imaging is the s-SNOM technique. It builds on atomic force microscopy (AFM) and adds to it optical spectroscopic capabilities. This is achieved by illumination of the AFM probe tip by a tightly focused light beam and detection of the light scattered from the apex of the tip. Both cantilever-type [288] and tuning-fork-type [289] probes are employed. The rise of s-SNOM began at the turn of the century [290,291] after a researcher had learned how to suppress the abundant background signal (by lock-in measurements referenced to the probe tip’s oscillation frequency), how to extract the information from the tip apex alone (by higher-harmonic demodulation), and how to achieve sensitivity to both the amplitude and phase of the scattered radiation and simultaneously to amplify it (by coherent techniques [292], such as the now dominant pseudo-heterodyne detection [293]).

It is worth mentioning that s-SNOM is not purely surface-sensitive, but probes a volume down to about 50 nm in dielectric media [294], which gives it tomographic capabilities [295,296,297,298]. This capability is still to be exploited in the THz frequency regime, e.g., for the (also quantitative) characterization of electrically conductive layers buried under a dielectric layer [299].

In the last two decades, developments have focused on the IR spectral regime. The THz regime has seen some early work [273,300,301], but only recently has found increased attention [302]. This delay is related to the usual challenges of THz sensing and spectroscopy, such as the large size of focal spots, the limited power availability, the limited choice of suitable detectors, and the need to cryogenically cool the most sensitive types of them. However, with the development and adaptation of dedicated THz technologies, this deficiency is currently being removed, and it is easy to foresee the advent of fascinating spectroscopic imaging studies.

Important THz-specific technological advances have already taken place. With regard to the probe tips, longer and more resonant tips, which exhibit a stronger field-focusing and scattering efficiency, have been introduced [303,304,305,306]. In the course of the investigation and optimization of the probes, researchers also turned away from the cantilever-type probes of AFM and explored probes based on quartz tuning forks [289,299,306,307], as they are used for STM. Unlike cantilever-based probes, the needle is not an integral part of the probe, but attached separately to the tuning fork, which makes it easier to select a suitable length of the tip and to also work with different kinds of tip materials, e.g., indium [306], instead of the more common tungsten. The best lateral spatial resolution was reported to be <15 nm with a very sharp, cantilever-based tungsten tip with a radius of curvature of *R* = 6 nm, operated at a laser wavelength of 2.52 THz [305]. Even better resolution down to 5 nm (as achieved in the IR with a tip with *R* = 3 nm) may be possible [308]. The tapping amplitude had to be kept smaller than *R* for this resolution. As impressive as this is, it seems that atomic resolution, which AFM itself is capable of, is not attainable with s-SNOM.

Under routine operation conditions, THz s-SNOM reaches the same resolution of typically 50 nm as s-SNOM at shorter wavelengths [302]. A surprising finding of the study of Reference [305] was that blunt tips yield much higher s-SNOM signals than sharp tips of the same length (clearly at the price of a reduced spatial resolution). This is not obvious, per se, because the field enhancement at the tip is reduced; however, it is found to be overcompensated by the linear increase of the induced dipole moment with the value of *R*.

**Figure 6:**
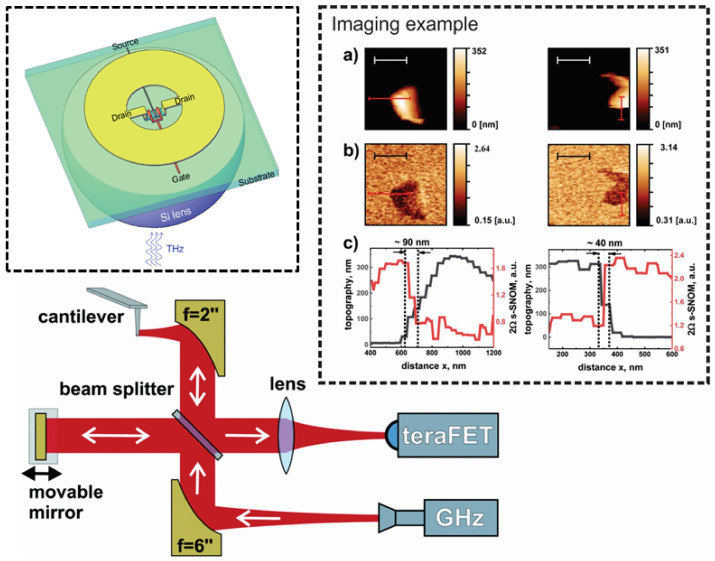
Schematic of an all-electronic homodyne s-SNOM measurement setup with a detector based on a Si CMOS field-effect transistor. Left inset: 3D layout of the detector with the monolithically integrated annular ring antenna and the Si substrate lens. Active devices were fabricated using either a 90 nm or a 180 nm technology process node. The high sensitivity enables demodulation of the s-SNOM signal at up to the 10th harmonic of the cantilever’s oscillation frequency. Right inset: (**a**) AFM topography of a Si surface (black) with dielectric islands (brown); (**b**) simultaneously measured s-SNOM amplitude images recorded at the 2nd harmonic of the cantilever’s oscillation frequency. Scale bars in (**a**,**b**): 1 µm. (**c**) Line scans along the red lines in (**b**) showing a spatial resolution down to <50 nm. Figure modified from Ref. [318].

With regard to radiation sources and detection, researchers employ free-space broadband TDS measurement systems [299,303,306,309,310], with the s-SNOM setup often implemented in such a way that pre-alignment is possible with the laser beam used for the generation of the THz pulses. In addition, integration of a photo-switch onto the AFM cantilever has been demonstrated for improved coupling to the tip [311]. Concerning narrow-band sources, most of the known coherent THz emitters seem to have been employed already, from large-scale devices, such as free-electron lasers [312,313] over molecular gas lasers [304,305,308,310], to compact sources, such as quantum cascade lasers [289,314] and electronic multiplier chains [315,316,317,318]. Frequency coverage by the latter was demonstrated up to 0.75 THz [315]; either uncooled Schottky diodes [315,316,317] or nanometric FETs (TeraFETs) [318] were used as detectors, making the corresponding s-SNOM systems all-electronic measurement instruments. Figure 6 displays such an instrument, a homodyne measurement setup of Reference [318], together with AFM and s-SNOM measurement results. While the latter were obtained with radiation at 246.5 GHz, TeraFETs equipped with various antennas can cover the entire THz frequency range (however, not with one single device) and in combination with various radiation sources [152,319]. They can be fabricated in various material systems, such as AlGaN/GaN [320,321], or, as here, be foundry-produced entirely in mainstream Si CMOS technology [150].

Typical applications of THz s-SNOM are the discrimination of materials via their spectral characteristics [308], which include the spectroscopy of phase transitions [322], and the tracing of surface waves, such as plasmon polaritons [313]. Until now, most studies are limited to solid-state materials. It should be mentioned that the microwave spectral region—due to the need for circuit diagnostics—has also seen much activity in the realm of near-field sensing techniques. The s-SNOM-related techniques applied there are known under the name Scanning near-Field Microwave Microscopy (SMM) [323,324]. Commercial products are available, e.g., from Keysight Technologies.

**THz STM:** The best spatial resolution is achieved in experiments, where the specimen is excited by THz radiation and the ensuing nonlinear response is measured via the changes of the tunneling current through an STM. In order to achieve appreciable effects, such experiments are performed with intense THz pulses. In contrast to STM combined with optical excitation at shorter wavelengths, where thermal effects play a significant role [325], it was found that the use of THz pulses does often not affect the measurements in a critical way [326]. This turned out to be an important observation especially for time-resolved experiments, which, if performed with short-wavelength pulses, are severely hampered by artefacts. The finding triggered a flurry of research activities which soon led to the mapping of the vibrational response of a single molecule (pentacene) with atomic spatial resolution [327], and to the control of the tunneling current through single atoms on Si surfaces [328]. The technique itself and its applications are now being explored intensively [329,330,331,332]. Even excitation at shorter wavelengths has recently been studied again with IR pump, THz probe experiments on C${}_{60}$ multilayer structures [333]. One can expect many fascinating results in the future.

*Current challenges and possible future trends*: The action range of the THz near-field techniques discussed above is solid-state physics, materials science and electronics; in the life sciences, they play no role, or, at best, a marginal role, to date. While first attempts are being made to explore the application of s-SNOM on alive matter in biological contexts [334,335], this endeavor poses a tremendous challenge for s-SNOM and the other techniques. The reasons are obvious: The strong absorption by water and the character of alive matter as soft matter. It is not unlikely that, in analogy to AFM-IR, an AFM-THz technique will develop, characterized by an AFM tip being placed onto a biological specimen which is excited by THz radiation; the probe tip will then sense and map the mechanical changes induced by the the excitation.

Another limitation of the techniques discussed above is that they are essentially surface-sensitive techniques (even though s-SNOM has a depth sensing character to it, which, however, is restricted to the vicinity of the surface). What is missing is THz imaging technique which can probe tiny volumes in the depth of a specimen, especially of biological origin. If one considers sub-wavelength volumetric imaging techniques in the VIS spectral regime, with their enormous driving force being applications in cellular biology, one finds the best spatial resolution to be reached with two families of techniques, Stimulated Emission Depletion (STED), on one hand, and the Stochastic Optical Reconstruction (STORM) with its variants, such as Photoactivated Localization Microscopy (PALM), on the other. Both classes of techniques were honored in 2014 with the Nobel Prize, awarded to Stefan Hell for STED and to Eric Betzig and William Moerner for STORM/PALM, with the prize motivation “for the development of super-resolved fluorescence microscopy” [336,337,338]. They both reach a spatial resolution on the scale of 10 nm, enabling the observation of large proteins. And this resolution is obtainable within the volume of the specimen, not only at a surface. The success of these techniques is intimately linked with the availability of fluorophores which can be incorporated (often by genetic engineering techniques) into target areas of the cells. The techniques have hugely benefited from the discovery of the famous Green Fluorescent Protein (Nobel Prize 2008), which played and—with its derivatives—continues to play a key role for super-resolved fluorescence microscopy, but the application areas could be widened much with a broader portfolio of fluorophores [339].

It has been a dream of many to also adopt such concepts at THz frequencies. There are two fundamental challenges here. The first one relates to the availability and the operational conditions of power detectors with a useful response to single or only a few THz photons. One would expect that superconducting detectors are capable of such performance, but their single-detection capability, for various reasons, is limited to shorter wavelengths [340,341]. Single-photon detection is possible with cryogenically cooled quantum-dot detectors (which use a single-electron transistor as sensor for the polarization of the dot) and with charge-sensitive IR phototransistors (using quantum wells sensed by a 2DEG) [340]. New types of detectors, exploiting novel material systems, such as a magic-angle bilayer graphene, are under development [341]. However, the THz frequency range is affected much more severely than the IR or VIS ranges by ambient radiation, and, to have a “THz-dark” environment means to place the experiment with the specimen entirely into an ultracold cryostat, which is not very conducive for experiments on living cells, but at least experiments on dead biological matter are possible under such conditions. It is an interesting question, whether heterodyne techniques may develop a sensitivity sufficient for single- or few-photon detection.

The second challenge is the availability of fluorophores. We are not aware of any such compound for THz frequencies. One has to note that a fluorophore is an emitter with a delayed and repeated emission of radiation after excitation. A fluorophore has to be distinguished from contrast agents which are under investigation for the THz regime (and very much so for microwaves), with much work devoted to plasmonic metallic nanoparticles, carbon nanotubes, and graphene [342], with hyperthermia effects being one driving force. This lack of fluorophores implies that future THz techniques inspired by STED or STORM/PALM have to adapt to other emitters available at THz frequencies, of which contrast agents and amplitude/phase masks may be an option [266]. A question is to what extent these are able to enhance the spatial resolution [266]. Ideally, the emitters should be point-like sources, which seems to exclude objects with geometrical resonances (although antennae made from carbon materials have a length significantly less than half the wavelength at resonance [343,344]).

## 9. Advanced Specialized THz Imaging Techniques

### 9.1. THz Light-Field Imaging Technique

*Recent advances*: The light-field method relies on the idea to record both intensity and direction of the incoming light rays. This highly versatile computational method is well-known in visible light in enriched new facilities of synthesizing novel imaging views [345]. The light-field imaging in THz range was demonstrated using point THz source (hetero-barrier transistor array module consisting of 4 × 4 incoherent source pixels operating at 0.53 THz) and silicon hyperhemispherical lens integrated with 32 × 32 pixel CMOS camera which was used as the THz light-field sampler [346]. It was demonstrated that the light-field THz imaging can be a useful technique for radiation pattern characterization, and it also can serve as an option to record images in transmission mode without external focusing optical elements.

*Current challenges and possible future trends*: The realization of light field imaging in THz range using compact electronic transistor-based arrays can allow for reconstruction of 3D image using computer algorithms. In addition, using shape-from-focus method, one can collect additional information about the object along the depth dimension, which can be attractive for medical and other applications. However, the image recording in reflection mode, when the surface is rough, causes tremendous difficulties [345], hence needing special technological and computing approaches. As a result, compact near-field THz imaging systems are currently getting more preference, for instance, in biomedical applications [347].

### 9.2. Homodyne Spectroscopy and Phase Sensitive Interferometry

*Recent advances*: Phase-sensitive detection provides the possibility to enhance a detected signal by orders of magnitudes by a proper selection of its phase difference in respect to a strong local oscillator either in heterodyne or homodyne operation mode. Homodyne scheme does not require two emission sources synchronized via phase-locking loop and, thus, can be more attractive in comparison to heterodyne. Recently, the theory of homodyne phase-sensitive THz spectrometer based on nanometric FETs was developed, revealing that the FET can be used as a sensitive spectrometer and/or interferometer when exposed to a strong tunable local oscillator signal with the varying frequencies and phases [348]. The gain over 100 times was predicted, while maximum gain in the order of 10${}^{5}$ can be reached in a detection scheme involving the interference of a weak incoming signal and a strong signal of a local oscillator with the close frequency. Very recent experimental evidence supporting this theory employing a THz detector based on 90 nm Si CMOS FET and broadband bow-tie antenna was presented [151]. It was demonstrated that by employing a homodyne detection system overlaying the radiation from two photomixers, the SNR can reach up to 70 dB at the same frequency with an integration time 100 ms. This improvement and the spectroscopic evidence for water vapor lines was demonstrated up to 2.2 THz, indicating the potential of these detectors in practical applications of continuous wave THz spectrometry systems.

Phase-sensitive detection can be realized in graphene FET connected to specially designed antennas [241]: Here, the helicity- and the phase-sensitive conversion of circularly polarized radiation into dc photovoltage is caused by the plasmon-interference mechanism of two plasma waves in the channel, excited at the source and drain part of the transistor. The suggested plasmonic interferometer is capable of measuring the phase difference between two arbitrary phase-shifted optical signals. The observed effect opens a wide avenue for phase-sensitive probing of plasma wave excitations in two-dimensional materials.

*Current challenges and possible future trends*: Aforementioned techniques were developed for spectroscopy aims, and their extension and adoption in compact phase- and polarization sensitive THz imaging systems would be an important step in their broadening facilities, particularly in development of systems equipped with all-electric detectors for determination of THz radiation polarization state.

### 9.3. Room Temperature THz Comb Spectroscopy

Realization of optical frequency comb using QCLs paved a novel route for the development of highly sensitive compact spectroscopic systems [349,350]. An attractive application of optical frequency combs is the so-called dual-comb spectroscopy: here, multi-heterodyne detection is performed, allowing for Fourier transform spectroscopy without moving parts, while still providing high resolution and high sensitivity—several orders of magnitude higher than traditional Fourier spectrometers [351,352,353].

**Figure 7:**
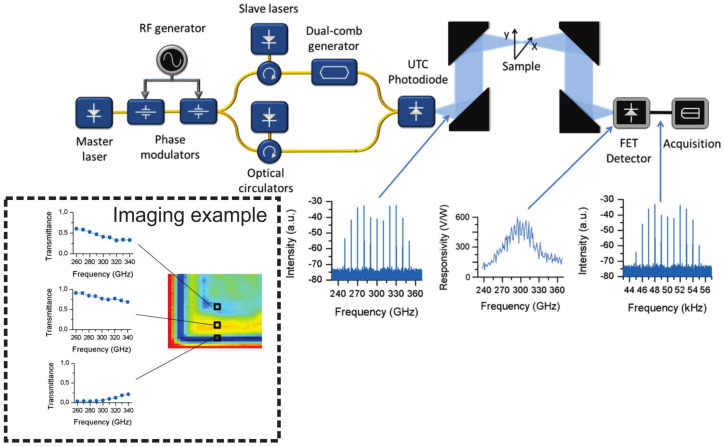
Block diagram of the THz dual-comb imaging system. The first optical frequency comb is generated from the output of the master laser by two phase modulators, and then two teeth (with a frequency spacing equal to the central THz signal to be generated) are filtered by optical injection locking and a dual-comb signal is created from one of them; both signals are recombined on an uni-traveling-carrier (UTC) photodiode. The emitted THz signal is focused on the sample plane and detected later by a FET detector. The insets show (from left to right) the spectrum of the THz dual-comb, the responsivity of the FET detector and the spectrum of the multi-heterodyne signal after detection. The imaging example presents the transmittance spectra through the complex plastic sample at several locations, enabling a straightforward separation between plastic layers (differences in the overall absorbance and the frequency dependent slope) and the edge diffraction. Reproduced from Ref. [361].

*Recent advances*: The demonstration of dual-comb spectroscopy in the mid-infrared QCLs frequency combs unveiled a potential of the approach as the basis for a compact and all solid-state-based broadband chemical sensing [354]. Relying on recent advances in this dynamic field of optical spectroscopy in the infrared and THz ranges [355], on-chip dual-comb source at THz frequencies at 23 K was designed and fabricated [356]. Here, the multi-heterodyne beating signal of two free-running THz quantum cascade laser frequency combs was measured electrically using one of the combs as a detector, and up to 30 modes can be detected corresponding to a spectral bandwidth of 630 GHz. Full phase-stabilization of a cryogenically cooled QCL-based comb against the primary frequency standard was demonstrated [357], proving independent and simultaneous control of the two comb degrees of freedom in modes spacing and frequency offset. Optical frequency comb synthesizer over the whole laser operational regime, with up 36 optically active laser modes delivering about 200 µW of optical power per optical mode was also shown [358]. As a significant breakthrough, a room-temperature THz harmonic frequency comb in 2.2 to 3.3 THz based on the difference-frequency generation from a mid-IR QCL has been considered [359]. In this design, THz harmonic-state frequency comb was intracavity generated via down-converting a mid-IR comb with an integrated mid-IR single mode based on distributed-feedback grating without using external optical elements. As a confluence of different technological concepts, it is worth noting a hybrid THz imaging system relying on THz QCL frequency comb and 100-pixel CMOS imager for the range of 3.25–3.5 THz with mode spacing of 17 GHz [360]. This approach has created a strong background to leverage both THz QCL and CMOS technologies to demonstrate new technological advances in THz imaging. The another interesting unification of technologies was demonstrated very recently by combining a novel THz hyperspectral imager based on an electro-optic THz dual-comb generator and a THz FET signal detector based on 90 nm CMOS technology [361]. The block diagram of the setup is presented in Figure 7. The image is acquired using raster scanning with a tailored optoelectronic dual-comb, enabling the frequency-multiplexed spectral characterization of 2D samples with the absolute frequency accuracy, as well as adjustable resolution and span. A resonant FET-based THz detector operating at around 300 GHz with an integrated slot antenna with impedance-transforming dipoles was used for the optimal detection of the THz dual-comb signal. The information on spectra taken with 10 GHz frequency resolution, as shown in the imaging example on a complex plastic sample, enables a straightforward separation between the plastic layers (differences in the overall absorbance and the frequency dependent slope) and the edge diffraction.

*Current challenges and possible future trends*: Despite a rapid evolution both in quantum and photonic THz-comb generation technologies, challenges remain in achieving broad spectral spans and the flat spectral intensity distributions. Compact solutions using QCLs in gas and condensed-phase spectroscopy revealed the potential of frequency comb and dual-comb spectroscopy approaches. The implementation of these principles in compact THz imaging can stimulate technological developments in on-chip-scale multispectral imagers for materials discrimination, biomedicine, and security application, as well as their implementation in environmental monitoring.

### 9.4. THz MCW Imaging

MCW stands for *modulated continuous wave* and denotes a family of techniques known from radar to determine distances [362]. This is achieved by amplitude or frequency modulation of the emitted carrier wave, followed by mixing of the signal reflected from the object with that generated at the moment of return. The difference frequency of the two waves then can be evaluated to find the distance. A common variant of the technique is frequency modulation (FMCW) with a linear chirp on the carrier wave.

In the THz regime, above 300 GHz, the exploration of FMCW saw a high tide ten to fifteen years ago, driven by the goal to develop active imaging systems for stand-off security applications [363,364,365,366,367]. While short-distance security imagers, such as those installed in screening portals at airports, obtain good spatial resolution with radiation at tens of GHz, because they can employ synthetic-aperture radar (SAR) techniques over a very large solid angle [368,369], the working conditions of stand-off imaging (with target distances from five to tens of meters) are much less favorable, as usually only a small numerical aperture is possible. The need for sufficient spatial resolution enforced the use of high radiation frequencies. Systems were developed and tested with carrier waves up to 812 GHz (with a single emitter, but eight detector channels [367]), frequency-limited by the available power and the increased losses incurred upon the beam propagation and the interaction with the objects-under-test. The use of the FMCW technique led to impressive results with regard to the detection of objects hidden under clothes against the clutter caused by scattering upon penetration of the radiation through the fabrics [364]. The ability to discriminate between object signals and clutter can be understood by considering that FMCW detection is the frequency equivalent of time-of-flight measurements, allowing for identification of objects in opaque media in a similar way as does OCT (optical coherence tomography [370]). Still, the limited image frame rates resulting from the single-beam raster-scanning approach in combination with prohibitive system costs associated with the delivery and handling of the high-frequency radiation prevented a breakthrough of these techniques to practical security applications. A challenge not to be underestimated is also the specular character of the reflectivity of the human body at THz frequencies, which makes object identification challenging [371,372].

The FMCW technique had more and continued success in its use for non-destructive testing, where it allows tomographic imaging of THz-transparent media. This application was pioneered by Synview GmbH, founded in 2007, which developed scanners at several frequency bands from 0.1 THz up to 0.8 THz [365,367]. The company went out of business in 2013, after transferring its technology to Becker Photonik, where it finds application and continued development in the frequency region 0.1–0.3 THz [373]. This regime appears to be generally most useful for non-destructive testing, mainly because clutter by background scattering from inhomogeneities of the material and its microstructure (such as in woven compounds) does not disturb the useful signal too much.

*Recent advances*: In order to improve the security scanners, Synview GmbH adopted multistatic/MIMO radar concepts in the THz frequency regime. A near-real-time FMCW-SAR imager with up to eight transmitters and 16 receivers working in the 220–320 GHz frequency range are reported [372,374]. It combined SAR imaging along a horizontal line and mechanical line displacement with a rotating cylindrical mirror in the vertical direction. The system was revolutionary in the sense that all emitters could fire simultaneously (instead of consecutively as in previous systems). The system was capable of a data acquisition speed of 1 ms per line, the frame rate then dependent on the rotation speed of the mirror. However, the system did not yet represent a breakthrough because the SNR and the channel discrimination were too poor. Other approaches aim at the combination of active and passive imaging techniques in order to overcome by sensor fusion the limitations of each technique itself [375].

In addition, with a view towards multi-channel systems, the Jet Propulsion Laboratory in the USA and others embarked on a large development effort devoted to Si micromachining [376]. Unlike the traditional milling-based fabrication process of hollow metal waveguides, which is sequential and time consuming, micromaching, which employs lithography and chemical etching, is a fast parallel process. In addition, it allows for the implementation of many new concepts of 3D stacking and interconnects not accessible with conventional techniques. It has to be seen how these advances will affect FMCW imaging.

With regard to the inspection of materials and objects, FMCW techniques at the interface between the microwave and THz frequency regimes have established themselves by now [377,378,379,380], and the measurement systems continue to be improved [381,382,383,384,385].

*Current challenges and possible future trends*: Concerning security scanners, which seem to be little explored until now, are distributed MIMO systems, with emitters and sensors spread out over a large range of angles around the region of interest.

Concerning all kinds of applications, sensor fusion is still in its infancy. Furthermore, as the hardware aspects mature, smart image acquisition and processing techniques, e.g., employing sparse imaging techniques, become more significant [378,386,387,388].

### 9.5. Passive THz Imaging

Imaging with (thermal) radiation emitted from the object-under-study or its surroundings avoids the use of additional active illumination. The concept is best known from radioastronomy, where usually superconducting receivers are employed for heterodyne detection with ultimate sensitivity. Passive detection was then also found to be very interesting for security scanners with microwaves and THz radiation. First, one avoids the costs of the active illumination, and, second, the thermal radiation penetrates the region of interest omnidirectionally, which immediately solves the problem of incomplete object images which one obtains with active systems because of the specular reflection characteristics of skin [371]. However, objects hidden under clothes on the body may have a similar temperature as the body itself, and then one relies on differences in the emissivity or on different coefficients of reflection of ambient radiation for their detection. Phase information is not available for depth resolution.

Passive imaging for security purposes made a major step forward at about the turn of the millenium, when HEMT-based low-noise amplifiers in GaAs, and especially InP technology, became available for frequencies up to 100 GHz. They made it possible to amplify the weak thermal signals over a large bandwidth with a gain of 50 dB [389]. A number of stakeholders developed mechanically scanned stand-off imagers, among them the British company Qinetiq which had systems working at 35 GHz and later at 94 GHz [390]. The famous image of a man holding a knife concealed in a newspaper was taken with such a system (see image, for example, in Ref. [391]). The scene was recorded outdoors and benefited from microwave illumination from the sky.

These technical developments (combined with rising fears of terrorist acts) triggered intensive research into stand-off security scanning (active and passive) in the decade around the year 2010. With the advance of microelectronic amplifier technology, it was shown that direct detection of thermal radiation is possible even at the remarkably high frequency of 670 GHz. This was demonstrated with an InP HEMT amplifier and a zero-bias Schottky diode as the detector. Measurements on a heated test object showed that a noise-equivalent temperature difference (NETD) of 2 K was reached (1/30 s integration time) [392]. For fast-scanning imaging systems, however, passive heterodyne detection was preferred. The company ThruVision introduced a system for imaging at 250 GHz with a bandwidth of 40 GHz [393,394,395] (using an 8-element receiver array; image frame rate: 3 frames/s [396]). Such a system was used in comparative passive security imaging tests in the VIS, IR and THz frequency regimes [394,397]. A heterodyne system operating at 640 GHz with a 20-GHz bandwidth was reported in Ref. [391], a 8-pixel system working passively at 250 and 340 GHz in Ref. [398].

A successful alternative to such photoelectric detectors are bolometers, devices exploiting the change of resistance of a conductor upon radiative heating [399]. Several types of such detectors are being used, respectively, and explored for passive THz detection.

- True real-time operation of security scanners was reached with passive imagers based on superconducting microbolometers. A system based on Nb transition-edge sensors (NEP on the order of 10${}^{-16}$ W/Hz${}^{1/2}$) was introduced by the Institute of Photonic Technologies, Jena [400]. In its final form, it had a 128-detector array and a main mirror with a diameter of 1 m, with the optical system designed for object distances of 15–20 m and image recording at a frame rate of 25 Hz with a NETD of 0.4 K [401]. The detectors were cooled to less than 1 K with a closed-cycle cooling unit. The system detected in a frequency band of 40–125 GHz around a center frequency of 350 GHz which was found to offer the best compromise between spatial resolution and fabric/clothes penetration [402]. It appears that system development is now being continued by Supracon AG. Another imaging system based on a superconducting Nb or NbN microbolometer arrays (64 elements), cooled to 4 K and operating at a scan rate of 5 fps with a NETD of about 2 K at a pixel integration time of 30 ms, was introduced by NIST (Boulder, CO, USA) in cooperation with VTT (Espoo, Finland) [403,404].
- Semiconductor bolometers: A much-used type of bolometer is the cryogenically cooled Si bolometer [399]. An extreme example is the ${}^{3}$He-cooled Si bolometer addressed in Reference [391]. It reached an NEP of 2 fW/Hz${}^{1/2}$, covering the frequency range 0.2–1.0 THz. Recent years have seen the development of devices for operation at room temperature, based on related advances of IR detectors. References [405,406] report about a membrane-mounted, antenna-coupled Si MOSFET bolometer which was fabricated by silicon-on-insulator micromachining techniques. An optical NEP of 7.8 pW/Hz${}^{1/2}$ and a NETD of 1.25 K for a 1-Hz effective noise bandwidth were measured. An array of similar passive Si MOSFET detectors for room-temperature operation was described in [407,408]. The detectors covered the 0.6–1.2 THz band. An optical NEP of 25 pW/Hz ${}^{1/2}$ was reached.
- Another important line of development for passive detection began, when the group of Qing Hu at the MIT reported in 2005 that microbolometer arrays, developed for room-temperature operation in the long-wavelength infrared (wavelength region: 7.5–14 µm), are sensitive enough to be useful for operation with QCLs emitting at 2.52 THz [184]. Cameras based on such arrays are commercially available, with a typical specification of the optical NEP of 0.9 pW/Hz ${}^{1/2}$ at their design wavelength. The arrays contain 2 × 10${}^{4}$–3 × 10${}^{5}$ detector elements, each consisting of a free-standing silicon nitride membrane with a vanadium oxide or other absorber, and a Si CMOS read-out [184]. The optical NEP at 4.3 THz was found to be 320 pW/Hz ${}^{1/2}$ [185], good enough to achieve >25 m stand-off imaging at the atmospheric window at 4.9 µm when measurements were performed with a powerful QCL radiation source [409]. Similar results were obtained at NEC, Japan, with their IR microbolometer cameras [410,411]. NEC introduced modifications to the camera system, replacing the camera window material and modifying the detector elements by adding absorber wings resonant at 3 THz [410,411]. This improved the NEP by a factor of 5–7, with an additional increase by pixel binning. LETI, Laboratoire d’Electronique, de Technologie et d’Instrumentation, of France, made even more rigorous changes to their IR microbolometer cameras and redesigned them for THz frequencies by the introduction of a bow-tie antenna and cavity [412].

*Recent advances*: Recent years have seen continued development of imagers, and progress here is closely associated with improvements of the detectors [413].

**Figure 8:**
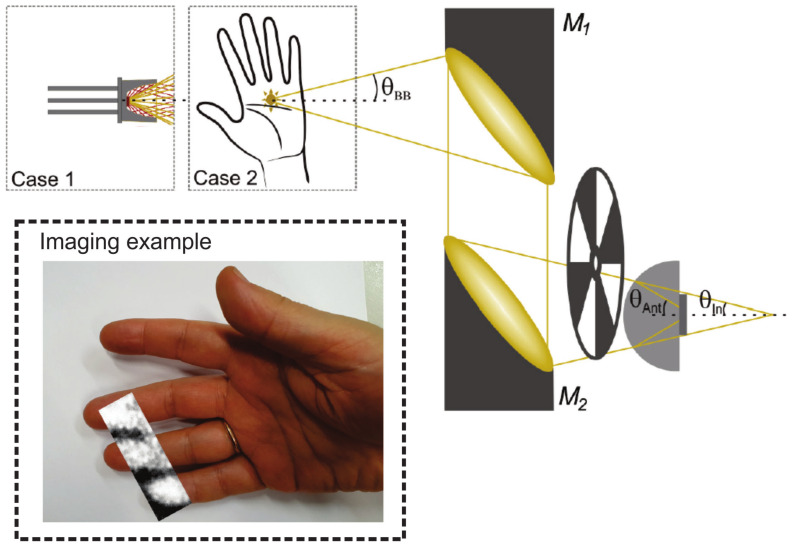
Detection of thermal radiation with a broadband nanometric FET [419]. The main panel shows the setup for the detection of radiation emitted from (**Case 1**) a ceramic heat source (embedded in a reflector) or (**Case2**) the human body, here, the palm of a hand. The heater, respectively, the hand, was placed on a xy-translation stage for raster-scan imaging. The radiation was guided by two parabolic mirrors and through a mechanical chopper to the detector, where it impinged via a Si substrate lens, attached to the backside of the nanometric FET chip, onto the antenna-embedded transistor. The angles shown are various acceptance angles used for the calculation of the expected noise-equivalent temperature difference (NETD). Inset: Raster-scan image of three fingers of a hand. The measurement was performed at ambient temperature. The black-and-white plot of the 25 × 75-pixel image (white indicating an elevated temperature; black representing room temperature) is superimposed on a photograph of the hand. Figure modified from Ref. [419].

- Superconducting systems have seen the development of arrays based on kinetic-inductance detectors. Two of their advantages are that they allow relatively easy up-scaling of the number of detector elements to form large arrays, and that operation of the NbN sensor elements is possible at somewhat elevated temperature—5–10 K—compared to transition edge bolometers. Ref. [414] reports development of such superconducting detector arrays for two frequency bands (0.1–0.45 THz and 0.45–0.625 THz). An unprecedentedly large number of 8208 membrane-mounted detectors was implemented on six tiles, forming an exceptionally large focal plane array with a diameter of 24 cm. A NETD of less than 0.2 K (with NEP values of the detectors on the order of 20 fW/Hz ${}^{1/2}$) is reported.
  A prototype of a security scanner, also based on kinetic inductance detectors, was recently introduced in Reference [415]. It has no mechanical scanning unit, but uses its 8712 detectors in a 20 × 10 cm${}^{2}$ array in “full-staring” mode. NETD values of less than 0.15 K/Hz ${}^{1/2}$ are reported (detector NEP: 14 fW/Hz${}^{1/2}$, detection up to 1 THz). The detectors were cooled to 5.8 K. Images were taken at 9 fps.
- Photoelectric rectifiers: The design of a 12-pixel array of Schottky diodes with differential read-out was introduced in Reference [416]. It is to capture blackbody radiation from 0.2 to 0.6 THz, and the pixels are predicted to have a NEP of 0.9 pW/Hz ${}^{1/2}$ and a sub-K temperature sensitivity.
  Direct power detection by distributive resistive mixing in antenna-coupled FETs was tested in several papers with regard to passive detection and imaging. Ref. [417] reported imaging with a 32 × 32 pixel Si MOSFET array and obtained a NETD of 21 K upon integration over 5.7 min at 30 fps. With antenna-coupled AlGaN/GaN HEMTs, cooled to 77 K and covering the band 0.7–0.9 THz, an NETD of 370 mK (0.2 s integration time) was obtained [235,418]. With an optical NEP on the order of 1 pW/Hz ${}^{1/2}$, the sensitivity was comparable with that of Si bolometers at 4 K. This allowed taking single-detector raster scan images, with a time of 20 min required for the acquisition of 5000-pixel images. Finally, Figure 8 displays an example of a raster scan image of the fingers of a human hand taken with a single antenna-coupled Si MOSFET held at room temperature [419]. The predicted (measured) NETD was 2.2 K (4.4 K) for a 1 Hz effective noise bandwidth (optical NEP of the detector: 42 pW/$\sqrt{\mathrm{Hz}}$). The temperature difference between the fingers and the ambient was 8.7 K, recording of the image took 30 min. The detailed analysis of these and related measurements confirmed that an improved detection of “grey”-body radiation can be obtained if the detector’s bandwidth is enlarged, and not if the corner frequency is shifted to higher values for a fixed bandwidth.
- Microbolometer arrays: The adaption of uncooled microbolometer arrays to THz frequency by the optimization of the cavity structures and antennas, respectively, metamaterial absorbers, as well as the read-out, continued to improve the sensitivity to a level that the cameras can be employed for passive imaging [420,421,422,423,424], albeit with a minimum detectable power of about 10 pW at 2.5 THz and a frame rate of 8 Hz [424] not for real-time passive imaging of the human body. Several companies are now on the market offering THz microbolometer cameras, for a list see Ref. [423].

*Current challenges and possible future trends*: Today’s imagers with superconducting detectors build on Nb or NbN technology. Although cryo-cooling has advanced enormously and closed-cycle cooling units avoid the costs for the delivery of cryogenic liquids, it seems that THz (security) scanners which require sub-10 K cooling are not accepted commercially. It is an interesting question whether detectors based on high-T${}_{C}$ superconductors [425,426] could lead to a change.

Radio astronomy remains to be a technology driver for heterodyne systems developments especially aiming for higher THz frequencies (mainly for gas spectroscopy) [427,428]. An example is the proposal to use graphene as the active mixer material for heterodyne receivers with quantum-limited sensitivity [429].

However, for the advancement of non-space applications of heterodyne detection—aiming at either fast, multi-pixel passive imaging or at coherent imaging, e.g., for holographic purposes—receiver arrays for operation at room temperature should be developed and be implemented in mainstream semiconductor technologies. Promising research is ongoing in this regard. A challenge is the LO power supply, with many issues, such as total power needed, on-chip power distribution, synchronization, and phase noise. The notion derived from space applications that one would need about 1 mW of LO power for each (Schottky-diode-based) mixer has been shown to be incorrect for THz imaging; much lower LO power levels permit coherent imaging and dynamic-range improvements over direct power detection [430,431]. Recent years have seen encouraging progress with regard to the development of scalable heterodyne detector arrays with distributed on-chip LO generation. A remarkable achievement in this respect is the implementation of a 32-receiver array in CMOS technology for detection at 240 GHz [432].

## 10. Artificial Intelligence in THz Imaging

Suitable quality for THz images of packaged objects or samples placed in optically scattering media, as a rule, becomes worse and a challenge to interpret, thus requiring either essential an increase of recording time, special means to subtract the background, or use an array of sensitive detectors. However, there is another—a “soft” way—to circumvent the aforesaid problems by application of artificial intelligence (AI) methods. In what follows, we shortly discuss AI-related routes dedicated for more effective operation of THz imaging systems.

*Recent advances*: Deep learning (DL) is a class of function interpolation algorithms which can be found to be especially attractive when the measurement is noisy, performed imperfectly in a real operational conditions, or image is recorded through a strongly scattering medium [243]. Additional factors can cause low transparency of many materials in the THz range, in particular, in medicine and security applications, making objects discrimination rather complicated. In these cases, DL can be applied not only to resolve objects but also to enable low-noise measurements and permit reconstruction from a single measurement [433]. Other AI tools, such as adjustable convolutional neural networks, can help alleviate the problem of a low spatial resolution in THz imaging [434]. In addition, DL can add new facilities for video sensing within the scope of snapshot compressive imaging when multiple high-speed frames are modulated by different coding patterns, then they are captured by a low-speed detector, and as captured measurement frame incorporates the information of all the coded frames, and reconstruction algorithms can be then employed to recover the recorded high-speed video [267]. The significant progress further is observed in DL-based photonic design by using various model architectures for specific photonic tasks [435].

*Current challenges and possible future trends*: One can expect further intensive implementation of DL and other AI instruments into THz imaging and spectroscopic systems [436], both for compact diffractive optics elements design, as well as improvements of recorded data quality. So-called ghost imaging can be one of such application areas [248]. The DL techniques can also pave the way to reach super-resolution in THz imaging as it evolves in widefield light sheet microscopy, for instance, using an Airy beam [437] or to remove non-resonant background as it was done in CARS experiments [438].

## 11. Summary, Systems Integration, and Possible Extrapolations in THz Imaging

High quality, effective, and reliable operation of room temperature THz imaging systems pose several challenging technological requirements. On the one hand, they should be compact and adhere to low power consumption, but, on the other hand, equipped with still powerful enough (in mW range) and preferably tunable THz emitter sources, compatible with diffractive optical elements and fast response (at least, in ns scale), and high sensitivity (in kV/W range and low NEP < 1 nW/$\sqrt{Hz}$) detectors operating over a broad (preferably, 0.1–10 THz) frequency range. Moreover, THz imaging systems require delicate balance between compact emitter power and detector sensitivity, as well as their overlap in the operational frequency range. Only fitting together all these requirements allows one to consider their combinations to construct compact and functional THz imaging systems.

**Figure 9:**
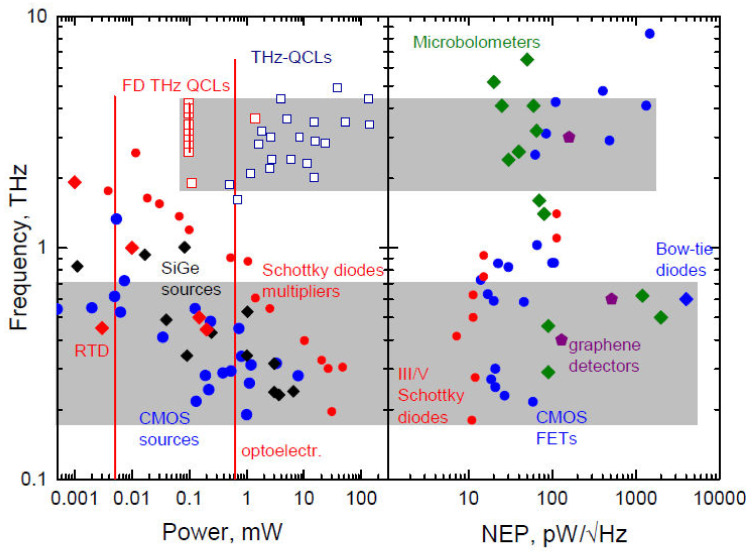
Map of room temperature emitting and sensing devices in a logarithmic THz frequency scale. The considered devices are plotted with respect to the emitting power in sources section (left) and the noise equivalent power (NEP) in sensors one (right). Taken data references: Schottky diodes multipliers [87,439] CMOS-based and SiGe-based electronic emitters [62]; CMOS and SiGe detectors parameters [159], parameters of Schottky detectors [440], microbolometers values [167], conventional THz QCL [41]; frequency-difference THz QCLs (FD THz QCLs) parameters—from publications by M. Razeghi [51,52] and M. Belkin’s [53] groups. Optoelectronic THz systems (denoted as red solid lines) are attributed for the emitters section only. The left red solid line depicts facilities of optoelectronic InGaAsBi-based systems [36], the right one—the system relying on InGaAs:Rh compound [37]. Resonant tunneling diodes data are taken from publications by M. Asada [119,121], M. Feiginov [117] and H. Yokoyama [441] groups. Shaded ares denote schematically possible complementary components in a design of compact THz imaging systems in respect to the THz frequency scale. One can note that compact room temperature THz imaging systems can be constructed, for instance, using FD THz QCLs and silicon nanometric transistors or microbolometers. It is seen that RTD devices can be used together with Schottky diodes, silicon nanoFETs, microbolometers and bow-tie diodes [181]. One can mention that CMOS technology-based mixers and oscillators in imaging can be nicely fitted together with nanometric FETs and microbolometers, as well as Schottky or bow-tie diodes. Graphene-based room-temperature THz detector’s parameters are taken from publications of the H. G. Roskos [163], J. Stake [442], and M. S. Vitiello groups [164].

Figure 9 presents a general map in the THz frequency scale displaying THz sources in regard to their emitting power and detectors in respect to their NEP aiming to fit them in design of compact room temperature operating THz imaging systems. Diffractive optics elements are not depicted as they can be used for the entire THz frequency range.

As one can see, the frequency scale mainly defines possible options in design of compact THz imaging systems, except optoelectronic THz set-ups (denoted as red solid lines) containing both optically-gated emitters-detectors pairs. It is seen that frequency-difference THz quantum cascade lasers-based compact imaging systems can be constructed only using silicon nanometric transistors or microbolometers down to the lowest reported QCL emission frequency of 1.9 THz. The RTDs can also reach this frequency, which can allow, in principle, their employment together with Schottky diodes, silicon nanoFETs, and microbolometers; however, the emitted powers at the highest—1.92 THz—operational frequencies are below 1 µW [121]. It is seen that CMOS technology-based mixers and oscillators gain a particular importance in subTHz frequencies providing reasonable powers and relatively wide freedom in design of compact imaging set-ups via fitting with nanometric FETs and microbolometers, as well as Schottky and bow-tie diodes.

**Figure 10:**
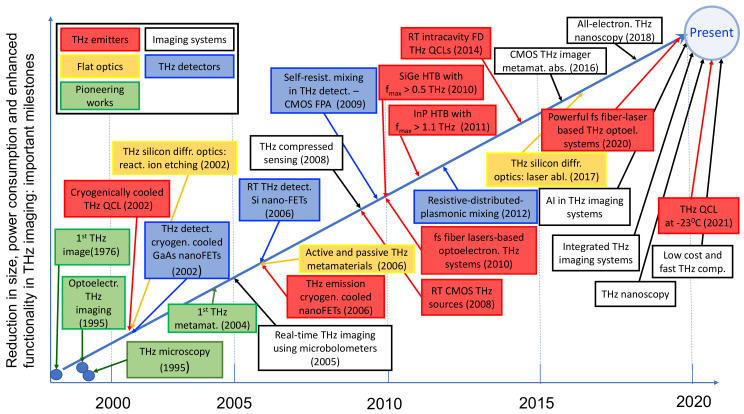
Schematic presentation of important milestones in the evolution of room temperature THz imaging systems within the last two decades. The systems are plotted with respect to the systems size reduction, power consumption, and enhanced functionality. Pioneering works in continuous wave [1] and optoelectronic THz imaging [2], as well as THz microscopy [273], and THz metamaterials [199] are denoted via blue circles and light-green labels. Meanings of other colors are depicted in the top-left corner of the plot. The first THz image recorded using optically-pumped molecular THz laser which is more than 2 meters long and uses of about 7 kW of electrical power, while state-of-art modern electronic sources, e.g., CMOS emitters are compact, in cm scale, and require only up to 1 W of power. No cryogenic cooling is needed for their operation. Invention of THz QCLs (still cooled cryogenically, below 50 K) unveiled an elegant solid-state-based compact solution for THz emitters—a route to reduce dimensions and power consumption in THz imaging systems [40]. First experiments on non-resonant THz detection using nanometric field-effect transistors open a new trend in development of sensitive detectors [139]. THz real-time imaging system using uncooled microbolometers array [184] demonstrated their potential for real time THz image recording. Room-temperature THz detection using silicon nanoFETs [141] was a breakthrough paper in the development of silicon-based THz and their sensors. Room-temperature generation of THz radiation in nanometric InAlAs/InGaAs and AlGaN/GaN HEMTs stimulated further research on nanotransistors. Self-resistive mixing mechanism in THz detection and development of CMOS technology-based THz focal-plane arrays provided a deeper understanding in physics behind the detection and opened a route for future cost-effective THz imaging solutions [144]. Femtosecond fiber lasers-based optoelectronics THz systems [35] demonstrated compact realization for optoelectronic THz imaging and increased convenience in their use. Room-temperature CMOS- [58,59,60], SiGe HBT-based [63] THz sources, and InP heterojunction bipolar transistors [64] revealed an interesting purely electronic approach to develop imaging systems. Resistive-distributed-plasmonic mixing models allowed gaining of wider insight into THz detection mechanism in nanometric FETs and extend their detection range up to 9 THz [148]. CMOS-based sensors and metamaterials absorbers can successfully be monolithically integrated [238]. Silicon-based diffractive optics can be a rational way for the integration of passive components [211,212] with active devices. Room temperature intracavity frequency difference tunable THz QCLs [51] exhibited a monolithic solution for THz spectroscopy, sensing and imaging systems. All electronic realization of THz nanoscopy allowed for creation of laser- and cryogenic cooling-free electronics-based near-field optical microscope [315]. Conventional THz QCLs has reached an operating temperature of 250 K, with the size of just a few millimeters [49].

Figure 10 manifests the most important milestones in evolution of room temperature THz imaging systems within the last two decades. The systems are plotted schematically in respect to reduction in THz imaging systems size, power consumption and enhanced functionality. The plot also indicates scientific breakthrough items in development of THz imaging systems and points out several dominating state-of-art activities, like development of low-cost and fast THz components, THz systems integration, and THz nanoscopy, as well as application of AI means in THz imaging. On the one hand, from the side of hardware, in particular, the trend of integration of constituents of the THz imaging system prevails in the development, thus allowing minimization of the system’s size and optical alignment issues, as well as develop compact room temperature operating THz sources and detectors. All these also enables to reduce strongly power consumption, enhance the functionality increasing accordingly the convenience in their practical use. For instance, the first THz image was recorded using optically-pumped molecular THz laser which is more than 2 m long and uses about 7 kW of electrical power, while modern state-of-art electronic sources, for instance, CMOS emitters are compact, only in cm scale, and require only up to 1 W of power. No cryogenic cooling is needed for their operation. This trend of room-temperature electronic THz oscillators, from initial works demonstrating their potential using CMOS-based transistors [58,59,60], heterojunction bipolar transistors (HBTs) of SiGe- [63] and InP-based structures [64], up to now, experienced essential progress. One can mention, for instance, highly linear ultra-wideband amplification with a gain bandwidth product of 1.75 THz [443] and mW range power in SiGe [444], respectively. The elegant solution to combine them for 3D imaging applications near 600 GHz using a set of oscillators based 250 nm InP HBT technology and a heterodyne image receiver based on 130 nm SiGe HBT technology has been demonstrated very recently [445].

A similar tendency can be seen in the evolution of femtosecond fiber lasers-based optoelectronics THz systems: Their emitted power has increased from several microwats [35] in 2010 to 637 µW in 2020 [37].

The conventional THz QCLs from cryogenically-cooled conditions have reached the operation temperature of 250 K with their length of a few millimeters [49], thus exhibiting a new level in cost, complexity, and portability of quantum THz emitters. It is of particular importance for direct implementation.

On the other hand, from the side of software, the installation of artificial intelligence in THz image processing and the simultaneous system integration create a wide basis for a new generation of THz imaging systems, making them compact electronically, “smart” computationally, and, hence, turning to the level of high quality standards already available in mobile phones’ cameras.

Concerning industrial applications of THz imaging and sensing, their possibilities were well-described recently [446,447]. Since industrial applications are very sensitive to convenience-in-use and reliability, we would like to underline only one technology which, in our opinion, can cause a breakthrough in industrial applications. The main development in industrial imaging applications should be brought by stand-alone CMOS sources and sensors that will allow developments in all-CMOS-based THz systems. One of the first such systems using Si CMOS MOSFETs was demonstrated in 2014 [157], where signal-to-noise ratio of 20 dB at 220 GHz using 50 ms integration time (equivalent noise bandwidth of 5 Hz) was achieved. In the next year, a compact multicolor THz imaging system with a chipset [448] was implemented in a 250-nm SiGe HBT BiCMOS process and operated in a heterodyne detection regime. Very recently, a 130 nm SiGe process was employed to build up a 420 GHz source system-on-a-chip and applied it for the computational imaging with a single-pixel camera and spatial modulation of the THz radiation [100]. A 60 dB voltage dynamic range was achieved for one 64-pixel image with a 100 ms acquisition time. These achievements have established a strong background that compact electronically-driven THz imaging technologies will be readily available for industrial needs, thus enriching its potential in more rapid high-tech evolution.

## Data Availability

Not applicable.

## Abbreviations

THz	Terahertz
THz TDS	Terahertz Time-domain Spectroscopy
MBE	Molecular Beam Epitaxy
SNR	Signal-to-Noise-Ratio
QCL	Quantum Cascade Lasers
RTDs	Resonant Tunneling Diodes
HEMT	High Electron Mobility Transistor
HBT	Heterojunction Bipolar Transistor
FET	Field Effect Transistor
HFET	Heterojunction Field Effect Transistor
CMOS	Complementary Metal Oxide Semiconductor
NEP	Noise Equivalent Power
MEMS	Microelectromechanical System
MMIC	Monolithic Microwave Integrated Circuit
TMIC	Terahertz Monolithic Integrated Circuits
CI	Computational Imaging
AI	Artificial Intelligence
DL	Deep Learning
